# Exploring the Phytochemical Profile and Biological Insights of *Epilobium angustifolium* L. Herb

**DOI:** 10.3390/plants14030415

**Published:** 2025-01-31

**Authors:** Reneta Gevrenova, Gokhan Zengin, Gulsah Ozturk, Dimitrina Zheleva-Dimitrova

**Affiliations:** 1 Department of Pharmacognosy, Faculty of Pharmacy, Medical University, 1000 Sofia, Bulgaria; dzheleva@pharmfac.mu-sofia.bg; 2 Physiology and Biochemistry Research Laboratory, Department of Biology, Science Faculty, Selcuk University, Konya 42130, Turkey; gokhanzengin@selcuk.edu.tr (G.Z.); gulsahhozturk97@gmail.com (G.O.)

**Keywords:** *Epilobium angustifolium* L., LC-MS profiling, antioxidants, enzyme inhibition

## Abstract

The aerial parts of *Epilobium angustifolium* L. (fireweed) (Onagraceae) are renowned for their use in the treatment of prostatic, kidney and urinary tract diseases, and skin infections. In this work, a comprehensive phytochemical profiling of the methanol-aqueous extract from *E. anfustifolium* aerial parts was performed by the means of liquid chromatography–Orbitrap high-resolution mass spectrometry. Annotation and dereplication of 121 secondary metabolites were achieved, including acylquinic acids, gallo- and ellagitannins, flavonoids, phenolic acids, and their glycosides. Forty-six compounds are reported for the first time in the species. Total phenolic and flavonoid content were 85.04 ± 0.18 mg GAE/g and 27.71 ± 0.74 mg QE/g, respectively. Antioxidant capacity assessment revealed that the extract actively scavenged DPPH and ABTS radicals (310.74 and 466.82 mg TE/g) along with a high reducing power in CUPRAC and FRAP assay (442.83 and 291.50 mg TE/g), respectively, and metal chelating (48.20 mg EDTA/g). The extract also had a distinct impact on α-glucosidase (3.48 mmol ACAE/g) and moderate activity towards α-amylase (0.44 mmol ACAE/g) and lipase (8.03 OE/g). It inhibited acetyl- and butyrylcholinesterase (2.05 and 1.67 mg GALE/g) and had a prominent anti-tyrosinase effect (61.91 mg KA/g). Our results contribute to establishing fireweed as a multifunctional agent for use in herbal preparations.

## 1. Introduction

*Epilobium angustifolium* L. (=*Chamerion angustifolium* Scop.) (Onagraceae) is renowned for its usage in nutraceuticals, diet supplements, and cosmetic products [1]. The species is distributed widely in the temperate zones of Europe, Asia, and North America [2]. *E. angustifolium* is commonly referred to as rosebay willowherb, fireweed, or great-willowherb [3]. It is a perennial plant with an erect stem up to 2 m high and which develops large racemes with a number of pink flowers [2].

Overall, more than 250 secondary metabolites, especially flavonoids, ellagitannins, and phenolic acids, have been reported in *E. angustifolium* extracts [1,2,4]. Fireweed also contains lignans, triterpenoids, steroids, fatty acids, essential oil, and alkaloids [1]. A high level of ellagitannins was determined, which represents about 15% of the dry mass of the herb [5]. Monomeric tellimagradin I and II along with macrocyclic dimer oenothein B and trimer oenothein A have been isolated from *E. angustifolium* aerial parts [5,6]. Oenothein B reached up to 50% of the total oligomeric ellagitannin level [6]. Its concentration varied from 2 to 4.5% in the raw plant material [7,8,9]. Flowers contained 10% more oenothein B as leaves, while the content of oenothein A was significantly lower [5]. In addition, gallotannins have been evidenced, including galloyl-glucose and galloyl-HHDP-glucose derivatives [10].

A variety of kaempferol-, quercetin-, and myricetin-glycosides has been reported, being present at 1–2% of the dry plant mass [1,3]. It is worth noting that the flavonol-rhamnosides have been found, especially in the flowers [5]. Recently, conjugates of flavonol glycosides with hydroxycinnamic acids have been reported (acylated flavonoids) [7]. Quercetin 3-*O*-glucuronide was the most abundant within the flavonol glycosides [11], reaching up to 4.75% [4,10]. Consequently, oenothein B and quercetin 3-*O*-glucuronide have been ascribed as marker compounds for raw material standardization [12,13]. Phenolic acids in the fireweed herb were delineated by gallic, ellagic, protocatechuic, caffeic acid, and acylquinic acid isomers [3,7,14]. HPLC-DAD-MS, HPLC-ESI-MS/MS, and UHPLC-DAD-ESI-QqQ-MS have been applied for identification/annotation and the quantitative determination of ellagitannins, flavonoids, and phenolic acids [5,9,10,14,15,16,17]. Interestingly, the inflorescence stem (from the apex) accumulated the highest content of total polyphenols (up to 250 mg/g dw) [5]. Oenothein B dominated these parts, being present at 148 mg/g dw. Quercetin 3-*O*-rhamnoside and kaempferol 3-*O*-rhamnoside reached 5.16 and 7.91 mg/g dw in flower buds, respectively, while they were not evidenced in the leaves [5].

*E. angustifolium* aerial parts have been used in ethnopharmacological approaches as an astringent and wound-healing agent, and for the treatment of skin infections, urinary disorders, and migraine headaches [1,2,13]. In Europe, the main use is to treat prostate inflammation, and kidney and urinary tract diseases [2]. The phytochemistry, pharmacology, and traditional usage of *Epilobium* L. species are the subject of literature reviews by Granica et al. [3] and Vitalone and Allkanjari [18] which strongly emphasize the anti-inflammatory potential of *E. angustifolium* extracts and the beneficial effects of plant formulations and oenothein B on benign prostate hyperplasia (BPH) and prostatitis, and the cytotoxic activity on human prostate cell lines. Adamczak et al. [2] summarized progress in phytochemical studies, and highlighted the variability of bioactive compounds in relation to plant parts, geographical origin, and phenological phases. In the review of Schepetkin et al. [13], particular attention was paid to the potential clinical use of the fireweed oenothein B, flavonoids, and other polyphenols. The effects of oenothein B on abnormal prostate cells, where it inhibits cell proliferation and prostate-specific antigen (PSA) secretion, sustain traditional claims relating to fireweed use for the mitigation of prostate issues.

Significant advances have been made in understanding the mechanisms of fireweed extract-mediated effects on BPH, which has been related to the modulation of prostate enzyme activity (5-α-reductase, aromatase, metalloproteinases), the regulation of androgen levels, the activation of the mitochondrial pathway of apoptosis, and pro-inflammatory cytokine production [13,16]. In addition to evoking an antioxidant response, oenothein B from *E. angustifolium* enhances the activity of various types of immune cells [13]. Herbal teas and dietary supplements from *E. angustifolium* are available in European Union countries [16]. In the monograph on *E. angustifolium* published by the European Medicinal Agency (EMA), these products are shown to be consistent with the requirements for “traditional use” (https://www.ema.europa.eu, accessed on 1 November 2024).

In monocentric, randomized, double-blind, placebo-controlled clinical trials, *E. angustifolium* food supplements, standardized to contain ≥15% oenothein B, improved the quality of life of subjects with BPH by reducing nocturia and aiding general renal function [16].

*E. angustifolium* aqueous extract did not lose its biological activity after in vitro digestion and did not affect intestinal bacteria growth [4]. Taking into consideration its cytotoxic activity towards colon cancer cell lines, the infusion has been evaluated as a supporting agent in colon cancer therapy. Dreger et al. [2] reviewed the antibacterial and antimycotic activity of fireweed extracts, emphasizing the potential use of the herbal drug as antimicrobial agent in wound healing, in cosmetic products, and in food preservatives.

It is worth noting that the aforementioned studies emphasize the main flavonoids and ellagitannins, while there is not a comprehensive metabolite profile of fireweed provided by means of liquid chromatography–Orbitrap high-resolution mass spectrometry (LC-HRMS).

The cited studies generate further interest in fireweed and have prompted us to undertake a comprehensive profiling of the secondary metabolites of methanol-aqueous extract from *E. anfgustifoliun* aerial parts by means of ultra-high-performance liquid chromatography–Orbitrap high-resolution mass spectrometry, integrated with an assessment of its antioxidant and enzyme inhibitory potential. Owing to the fact that crucial enzymes are considered to be targets in the pharmaceutical therapies, the enzyme inhibitory activity of the studied extract towards cholinesterases, tyrosinase, α-amylase, α-glucosidase, and lipase was determined. In addition, the evaluation of the antioxidant capacity via different mechanisms may have an important contribution in evoking an antioxidant response in the aforementioned conditions.

## 2. Results and Discussion

### 2.1. UHPLC-HRMS Profiling of Secondary Metabolites in E. angustifolium Extract

**Figure 1:**
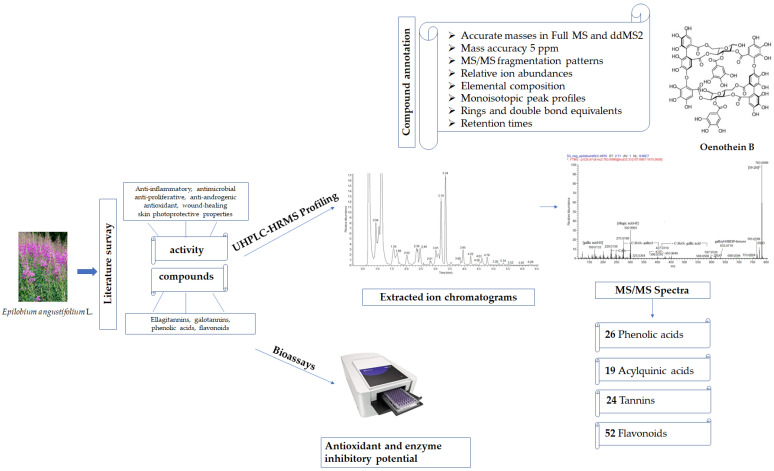
The workflow for the *Epilobium angustifolium* study.

An in-depth UHPLC-HRMS analysis of *E. angustifolium* methanol-aqueous extract was performed, allowing for the identification/annotation of 121 secondary metabolites (Table 1). The complete workflow, combining UHPLC-HRMS data annotation and biological potential, is presented in Figure 1. Identification confidence levels for metabolite profiling were performed according to Sumner et al. [19] and were as follows: level 1–compounds identified by comparison to the reference standard; level 2–putatively annotated compounds. Twenty-six compounds were assigned to phenolic acids. Nineteen compounds were ascribed as acylquinic acids. Twenty-four tannins and fifty-two flavonoids were also identified in the studied extract. Among all these 121 metabolites, 11 phenolic acids and derivatives, 10 acylquinic acids, 3 tannins, and 22 flavonoids are reported for the first time in *E. angustifolium* aerial parts.

#### 2.1.1. Phenolic Acids, Their Glycosides, and Coumarins

The phenolic acids frequently exist in the form of glucosides (the bond with sugars occurs via one of the phenolic functions) [20,21]. The basic compounds of the hydroxybenzoic acid series were gallic (**2**) and protocatechuic acid (**8**) and their hexosides (**3**, **5**, **6**), along with hydroxybenzoic, gentisic, vanillic, syringic, and hydroxyphenyllactic acid—hexosides (**4**, **7**, **9**, **11**, and **16**). The hydroxycinnamic acid series was represented by coumaric and caffeic acid-hexoside (**12**, **13**, **18**, and **22**) together with the respective free acids (**17**, **23**, and **24**). Based on the accurate masses and conformity of reference standard retention times and fragmentation patterns, **2**, **8**, **15**, **17**, **23**, **24**, and **26** were unambiguously identified in the studied extract (Table 1).

**Table 1:** Secondary metabolites in *Epilobium angustifolium* methanol-aqueous extracts.

No.	Identified/Tentatively Annotated Compound	Molecular Formula	Exact Mass [M-H]^−^	Fragmentation Pattern in (-) ESI-MS/MS	t_R_ (min)	Δ ppm	References
**Hydroxybenzoic, Hydroxycinnamic Acids, Phenylethanoid Glycosides, and Coumarins**							
1.	galloyl *O*-hexose	C_13_H_16_O_10_	331.0671	331.0676 (100), 271.0463 (10.2), 241.0349 (3.1), 211.0244 (10.4), 169.0133 (45.8), 151.0024 (13.4), 125.0230 (17.7), 107.0124 (4.7)	0.94	1.511	[16,17]
2.	gallic acid ^a^	C_7_H_6_O_5_	169.0142	169.0132 (33.9), 125.0230 (100), 107.0123	1.14	6.133	[10,16,17,22,23]
3.	gallic acid *O*-hexoside 1	C_13_H_16_O_10_	331.0687	331.0676 (100), 271.0465 (0.7), 241.0353 (1.9), 211.0246 (0.6), 169.0131 (16.7), 168.0054 (31.9), 125.0229 (36.7), 107.0123 (2.1)	1.17	1.511	
4.	hydroxybenzoic acid-*O*-hexoside	C_13_H_16_O_8_	299.0778	299.0765 (0.5), 137.0231 (100), 93.0330 (65.2),	1.26	−2.543	
5.	gallic acid *O*-hexoside 2	C_13_H_16_O_10_	331.0687	331.0674 (8.1), 169.0132 (16.7), 125.0236 (38.3)	1.56	1.118	
6.	protocatechuic acid-*O*-hexoside	C_13_H_16_O_9_	315.0727	315.0725 (100), 153.0181 (27.1), 152.0103 (56.8), 123.0072 (2.4), 109.0285 (9.9), 108.0202 (91.8), 81.0332 (0.6)	1.68	1.221	
7.	vanillic acid-*O*-hexoside	C_14_H_18_O_9_	329.0875	329.0903 (2.3),167.0339 (100), 152.0102 (24.0), 123.0437 (14.5), 108.0202 (39.9)	1.77	7.581	
8.	protocatechuic acid ^a^	C_7_H_6_O_4_	153.0181	153.0182 (15.4), 109.0280 (100), 91.0174 (1.2), 81.0331 (1.2)	2.02	−1.392	[14,23]
9.	*p*-hydroxyphenylacetic acid *O*-hexoside	C_14_H_18_O_8_	313.0932	313.0915 (1.6), 151.0388 (98.2), 123.0436(1.0), 107.0487 (100)	2.13	0.988	
10.	hydroxybenzoyl hexose	C_13_H_16_O_8_	299.0778	299.0774 (26.4), 239.0559 (25.4), 209.0447 (7.4), 179.0339 (28.6), 137.0230 (100), 109.0280 (23.3), 93.0329 (12.4)	2.16	0.600	
11.	syringic acid-*O*-hexoside	C_15_H_20_O_10_	359.0985	359.0991 (7.6), 197.0448 (100), 182.0212 (22.1), 166.9976 (8.3), 153.0545 (16.6), 138.0309 (28.5), 123.0073 (32.9)	2.26	2.311	
12.	caffeic acid-*O*-hexoside 1	C_15_H_18_O_9_	341.0871	341.0870(4.2), 179.0340 (100), 135.0438 (55.5), 107.0485 (0.3)	2.40	−2.332	[16]
13.	caffeic acid-*O*-hexoside 2	C_15_H_18_O_9_	341.0871	341.0865 (25.7), 281.0658 (2.3), 251.0563 (5.7), 221.0454 (1.9), 179.0341 (26.5), 161.0233 (100), 135.0439 (9.3), 133.0282 (26.4)	2.61	−3.827	
14.	caffeoyl-*O*-hexose	C_15_H_18_O_9_	341.0871	341.0859 (19.5), 281.0668 (93.4), 251.0561 (50.7), 221.0452 (47.1), 179.0341 (100), 161.0233 (6.2), 135.0438 (69.4), 133.0281 (20.4)	2.82	−5.469	
15.	4-hydroxybenzoic acid ^a^	C_7_H_6_O_3_	137.0230	137.0230 (100), 119.0123 (2.5), 108.0200 (9.8), 93.0329 (13.4), 65.0380 (1.1)	2.84	−10.052	[17]
16.	gentisic acid *O*-hexoside	C_13_H_16_O_9_	315.0727	315.0725 (7.9), 153.0181 (100), 125.0230 (5.1), 109.0279 (2.6), 81.0329 (0.4)	2.84	1.221	
17.	*p*-coumaric acid ^a^	C_9_H_8_O_3_	163.0389	163.0390 (6.2), 119.0488 (100), 93.0331 (3.6)	3.01	−6.792	[23]
18.	caffeic acid *O*-hexoside 3	C_15_H_18_O_9_	341.0871	341.0877 (27.3), 281.0663 (0.9), 251.0562 (2.2), 221.0456 (0.9), 179.0340 (100), 135.0438 (71.6), 107.0488 (0.5)	3.07	−0.602	
19.	methylgallate	C_8_H_8_O_5_	183.0299	183.0289 (100), 168.0053 (11.9), 140.0103 (11.4), 111.0074 (6.2), 83.0122 (0.7)	3.15	0.711	[14]
20.	quinic acid	C_7_H_12_O_6_	191.0549	191.0552 (100), 173.0447 (1. 8), 155.0336 (0.2), 127.0387 (4.1), 111.0435 (1.1), 93.0330 (6.6), 85.0278 (18.4)	3.19	−5.817	[16]
21.	umbeliferone	C_9_H_6_O_3_	161.0244	161.0233 (81.1), 133.0281 (100), 115.0173 (0.7), 105.0331 (1.5), 89.0381 (0.8), 77.0381 (0.8)	3.19	−7.250	
22.	coumaric acid-*O*-hexoside	C_15_H_18_O_8_	325.0930	325.0922 (2.3), 163.0389 (100), 145.0284 (0.3), 119.0488 (90.0), 93.0331 (0.3)	3.34	−3.355	
23.	caffeic acid ^a^	C_9_H_8_O_4_	179.0339	179.0341 (17.8), 135.0438 (100), 107.0488 (1.2)	3.54	−6.044	[23]
24.	*o*-coumaric acid ^a^	C_9_H_8_O_3_	163.0389	163.0388 (8.3), 119.0487 (100), 93.0330 (1.3)	4.55	−7.467	[23]
25.	galloyl-(caffeoyl)-hexose	C_22_H_22_O_13_	493.0988	493.1006 (100), 341.0881 (5.9), 281.0667 (32.9), 251.0560 (24.8), 221.0449 (13.9), 161.0233 (26.0), 135.0438 (31.4), 169.0132 (14.7), 125.0230 (10.4), 107.0123 (1.7)	4.78	3.643	
26.	salicylic acid ^a^	C_7_H_6_O_3_	137.0230	137.0230 (8.3), 108.0202 (7.5), 93.0330 (100), 65.0380 (0.7)	6.29	−10.125	
**Mono- and Diacylquinic Acids**							
27.	3-galloylquinic acid	C_14_H_16_O_10_	343.0671	343.0675 (38.9), 191.0554 (13.6), 173.0446 (22.2), 169.0132 (100), 125.0230 (32.6), 107.0124 (6.5), 93.0331 (7.8), 85.0279 (4.6)	1.25	1.370	
28.	neochlorogenic (3-caffeoylquinic) acid ^a^	C_16_H_18_O_9_	353.0867	353.0883 (39.5), 191.0552 (100), 179.0340 (62.7), 173.0446 (2.7), 161.0230 (4.1), 135.0438 (50.8), 111.0434 (1.6), 93.0331 (3.9), 85.0279 (8.2)	2.36	1.458	[5,10,23]
29.	3-*p*-coumaroylquinic acid	C_16_H_18_O_8_	337.0928	337.0934 (7.6), 191.0553 (14.4), 163.0390 (100), 135.0437 (0.6), 119.0488 (31.3), 111.0432(1.5), 93.0329 (2.6), 85.0278 (3.0)	3.01	1.096	[10,16]
30.	chlorogenic (5-caffeoylquinic) acid ^a^	C_16_H_18_O_9_	353.0874	353.0884 (4.2), 191.0553 (100), 173.0447 (1.1), 161.0235 (1.2), 111.0434 (1.5), 93.0331 (2.8), 85.0278 (2.0)	3.19	0.665	[10,16,17,23]
31.	4-caffeoylquinic acid	C_16_H_18_O_9_	353.0878	353.0882 (33.3), 191.0553 (43.1), 179.0340 (68.6), 173.0445 (100), 135.0438 (57.5), 111.0434 (3.6), 93.0331 (21.0), 85.0279 (7.8)	3.37	−0.100	
32.	3-feruloylquinic acid	C_17_H_20_O_9_	367.1034	367.1039 (18.2), 193.0497 (100), 191.0553 (2.6), 173.0445 (4.0), 149.0594 (3.4), 134.0359 (54.0), 111.0435 (1.5), 93.0330 (2.1), 85.0276 (0.4)	3.44	1.157	[10,16,17]
33.	1-galloyl-3-caffeoylquinic acid	C_23_H_22_O_13_	505.09	505.0999 (64.0), 353.0883 (38.8), 191.0553 (100), 179.0341 (52.5), 173.0443 (3.9), 161.0233 (4.2), 135.0438 (52.8), 111.0436 (2.0), 93.0330 (3.8), 85.0280 (8.0)	3.58	2.348	
34.	4-*p*-coumaroylquinic acid	C_16_H_18_O_8_	337.0928	337.0937 (10.2), 191.0551 (10.8), 173.0444 (100), 163.0390 (19.3), 119.0488 (14.8), 111.0430 (2.3), 93.0330 (18.7), 85.0279 (3.0)	3.79		
35.	5-caffeoylquinic acid isomer	C_16_H_18_O_9_	353.0874	353.0884 (6.4), 191.0550 (100), 179.0341 (0.9), 173.0446 (0.9), 161.0232 (1.9), 111.0436 (1.1), 93.0329 (2.5), 85.0279 (8.4)	3.88	1.684	[16]
36.	5-*p*-coumaroylquinic acid	C_16_H_18_O_8_	337.0928	337.0934 (8.1), 191.0552 (100), 173.0444 (7.1), 163.0389 (6.4), 145.0283 (1.2), 119.0487 (5.3), 93.0330 (19.0), 85.0278 (4.9)	3.95	1.629	[10]
37.	1-caffeoyl-3-galloylquinic acid	C_23_H_22_O_13_	505.0988	505.0999 (100), 353.0883 (5.7), 343.0675 (15.2), 191.0553 (26.5), 179.0342 (18.2), 173.0445 (18.2), 169.0123 (73.3), 161.0230 (6.4), 135.0440 (14.8), 125.0230 (33.1), 107.0124 (4.6), 93.0331 (8.5)	4.10	1.913	
38.	1-galloyl-5-caffeoylquinic acid	C_23_H_22_O_13_	505.0988	505.0998 (35.5), 353.0880 (18.2), 191.0551 (100), 179.0341 (3.1), 173.0447 (1.3), 161.0233 (2.4), 135.0437 (3.0), 111.0434 (0.9), 93.0331 (2.4), 85.0279 (6.9)	4.19	2.032	
39.	5-feruloylquinic acid	C_17_H_20_O_9_	367.1034	367.1040 (20.6), 193.0500 (11.3), 191.0553 (100), 173.0445 (49.6), 149.0598 (0.5), 134.0360 (15.5), 111.0437 (4.1), 93.0330 (29.2), 85.0279 (4.5)	4.39	1.402	[10]
40.	3-caffeoyl-5-galloylquinic acid	C_23_H_22_O_13_	505.0988	505.0996 (54.9), 353.0881 (71.0), 191.0552 (100), 179.0340 (56.8), 173.0447 (4.2), 161.0233 (4.7), 135.0438 (54.2), 111.0434 (0.7), 93.0329 (4.1), 85.0279 (8.4)	4.56	1.735	
41.	5-*p*-coumaroylquinic acid isomer	C_16_H_18_O_8_	337.0928	337.0935 (6.8), 191.0551 (100), 173.0443 (1.8), 163.0388 (1.8), 145.0279 (0.7), 119.0489 (1.1), 111.0435 (0.9), 93.0329 (4.8), 85.0279 (6.8)	4.61	1.926	
42.	caffeoyl-hydroxybenzoylquinic acid	C_23_H_24_O_12_	491.1196	491.1203 (94.4), 447.1305 (16.0), 329.0882 (0.9), 179.0340 (8.3), 161.0232 (100), 137.0235135.0438 (12.0), 133.0281 (40.6), 109.0281 (2.3), 85.0277 (1.5)	4.89	1.691	
43.	5-feruloylquinic acid isomer	C_17_H_20_O_9_	367.1034	367.1038 (9.5), 193.0498 (1.6), 191.0552 (100), 179.0341 (1.6), 173.0444 (2.3), 161.0229 (0.6), 134.0360 (2.8), 111.0434 (1.3), 93.0330 (6.3), 85.0279 (8.0)	4.91	0.939	
44.	3-galloyl-5-*p*-coumaroylquinic acid	C_23_H_22_O_12_	489.1038	489.1046 (44.3), 337.0936 (16.9), 191.0553 (2.9), 179.0704 (2.9), 173.0443 (1.5), 163.0397 (4.3), 146.0239 (2.7), 119.0488 (1.2), 111.0439 (0.9), 93.0327 (4.9), 85.0279 (6.8)	5.48	1.555	
45.	3-feruloyl-5-galloylquinic acid	C_26_H_32_O_11_	519.1872	519.1877 (100), 193.0498 (37.0), 178.0262 (16.0), 161.0235 (5.1)	6.70	0.954	
	**Tannins**						
46.	galloyl-HHDP-hexose 1	C_27_H_22_O_18_	633.0733	633.0746 (100), 300.9993 (73.0), 275.0201 (31.2), 249.0403 (22.4), 257.0088 (4.3), 245.0095 (2.5), 229.0141 (7.9), 185.0236 (4.6), 169.0132 (7.0), 145.0282 (1.9), 125.0231 (9.2), 107.0123 (2.5)	1.11	2.074	[10]
47.	galloyl-HHDP-hexose 2	C_27_H_22_O_18_	633.0733	633.0749 (100), 300.9994 (67.9), 275.0201 (36.5), 249.0405 (27.2), 257.0092 (4.8), 245.0085 (1.9), 229.0139 (8.7), 185.0235 (2.6), 169.0133 (8.5), 145.0282 (0.9), 125.0229 (9.5), 107.0124 (1.4)	1.40	2.264	
48.	digalloyl-hexose 1	C_20_H_20_O_14_	483.0780	483.0786 (100), 331.0677 (16.8), 313.0569 (10.1), 211.0240 (2.9), 169.0131 (81.5), 151.0021 (2.4), 125.0230 (56.8), 107.0124 (7.7)	1.60	1.266	[23]
49.	digalloyl-hexose 2	C_20_H_20_O_14_	483.0780	483.0786 (100), 331.0670 (9.0), 313.0570 (22.0), 271.0458 (7.4), 211.0243 (9.5), 169.0132 (49.9), 151.0026 (3.0), 125.0230 (30.8), 107.0125 (4.3)	2.60	1.204	
50.	oenothein B 1	C_68_H_46_O_44_	1568.1518 783.0686 [M-2H]^2−^,	783.0699 (100), 765.0590 (17.3), 633.0719 (1.0), 597.0530 (4.3), 427.0310 (3.2), 399.0376 (1.9), 300.9992 (27.7), 275.0199 (20.0), 273.0337 (0.4), 249.0405 (4.3), 245.0090 (4.6), 229.0139 (10.3), 217.0137 (3.7), 201.0186 (7.6), 169.0123 (9.5), 145.0282 (4.2), 117.0332 (1.6)	2.74	1.743	[5,10,16,17]
51.	digalloyl-HHDP-hexose (tellimagrandin I) 1	C_34_H_26_O_22_	785.0843	785.0864 (84.5), 300.9993 (100), 275.0200 (45.2), 257.0092 (6.6), 249.0409 (34.2), 245.0078 (4.1), 229.0141 (8.8), 201.0185 (5.5), 185.0232 (5.3), 173.0235 (3.6), 169.0133 (15.4), 145.0286 (3.4), 125.0231 (15.2), 107.0124 (3.2)	3.01	2.630	[10]
52.	digalloyl-hexose 3	C_20_H_20_O_14_	483.0780	483.0788 (100), 331.0680 (5.7), 313.0571 (17.7), 271.0462 (44.6), 211.0242 (13.1), 169.0132 (39.2), 151.0027 (1.9), 125.0231 (29.0), 107.0124 (4.3)	3.09	1.659	
53.	galloyl-HHDP- hexose 3	C_27_H_22_O_18_	633.0733	633.0747 (77.9), 463.0529 (3.4), 300.9992 (100), 275.0202 (11.1), 249.0406 (4.0), 257.0093 (4.7), 245.0095 (1.7), 229.0139 (7.6), 185.0232 (3.4), 169.0133 (2.3), 145.0285 (2.1), 125.0233 (2.9), 107.0121 (0.4)	3.24	2.169	
54.	oenothein B 2	C_68_H_46_O_44_	1568.1518 783.0686 [M-2H]^2−^,	783.0699 (100), 765.0596 (17.3), 633.0742 (1.6), 597.0522 (3.7), 427.0315 (4.5), 399.0354 (1.7), 300.9992 (27.7), 275.0198 (19.7), 273.0337 (0.4), 249.0402 (4.3), 245.0090 (4.6), 229.0139 (10.3), 217.0137 (3.7), 201.0186 (7.7), 185.0235 (2.4), 169.0133 (9.2), 125.0230 (8.2)	3.33	1.590	[10]
55.	brevifolin carboxylic acid	C_13_H_8_O_8_	291.0149	291.0151 (13.2), 247.0246 (100), 229.0170 (0.2), 219.0296 (3.1), 203.0341 (0.7), 191.0342 (9.3), 173.0234 (3.7), 145.0282 (3.4), 119.0487 (1.6)	3.34	1.511	
56.	digalloyl-hexose 4	C_20_H_20_O_14_	483.0780	483.0787 (100), 331.0675 (59.0), 313.0568 (10.8), 271.0462 (1.3), 211.0247 (1.6), 169.0133 (13.8), 125.0230 (45.6), 107.0125 (2.8)	3.35	1.390	
57.	oenothein A1	C_102_H_70_O_66_	2352.2277 1175.6083 [M-2H]^2−^,	1175.6090 (100) [M-2H]^2−^, 785.0854 (4.9), 765.0599 (9.2), 633.0746 (2.9), 597.0533 (3.1), 463.0533 (0.6), 427.0313 (4.0), 399.0364 (2.3), 300.9991 (35.7), 273.0043 (10.9), 257.0092 (4.6), 245.0090 (5.5), 229.0140 (9.9), 217.0138 (3.7), 201.0186 (7.3), 185.0235 (2.5), 173.0235 (4.4)	3.44	2.250	[5,15]
58.	digalloyl-HHDP-hexose (tellimagrandin I) 2	C_34_H_26_O_22_	785.0843	785.0865 (100), 300.9993 (96.9), 275.0199 (38.7), 257.0089 (5.8), 249.0403 (32.8), 245.0088 (3.2), 229.0135 (10.0), 201.0186 (5.7), 185.0236 (2.8), 173.0235 (3.6), 169.0135 (15.4), 145.0282 (3.4), 125.0230 (15.8), 107.0124 (3.2)	3.66	1.776	[10]
59.	oenothein A2	C_102_H_70_O_66_	2352.2277 1175.6083 [M-2H]^2−^,	1175.6084 (100), 785.0846 (3.9), 765.0589 (8.0), 633.0752 (3.3), 597.0527 (3.6), 463.0486 (0.7), 427.0307 (3.5), 399.0361 (2.0), 300.9990 (45.0), 273.0043 (9.6), 257.0092 (4.1), 245.0090 (5.6), 217.0135 (2.8), 201.0186 (7.4), 169.0133 (2.1)	3.89	1.731	
60.	trigalloyl-hexose 1	C_27_H_24_O_18_	635.0890	635.0904 (81.4), 465.0680 (100), 313.0569 (50.4), 253.0372 (0.8), 223.0259 (2.9), 193.0137 (5.5), 169.0132 (80.4), 151.0022 (3.7), 125.0230 (57.1), 107.0123 (12.0)	3.92	2.209	[10,23]
61.	trigalloyl-hexose 2	C_27_H_24_O_18_	635.0890	635.0905 (100), 483.0791 (11.1), 465.0680 (25.5), 313.0568 (27.7), 223.0236 (0.7), 193.0131 (4.9), 169.0131 (84.1), 151.0023 (1.8), 125.0229 (64.1), 107.0122 (7.1)	4.01	2.304	
62.	trigalloyl-hexose 3	C_27_H_24_O_18_	635.0890	635.0902 (100), 483.0786 (21.5), 465.0683 (11.3), 313.0568 (12.6), 253.0346 (0.4), 193.0134 (3.4), 169.0131 (49.2), 151.0024 (2.1), 125.0229 (49.7), 107.0123 (5.4)	4.11	1.926	
63.	trigalloyl-hexose 4	C_27_H_24_O_18_	635.0890	635.0905 (100), 483.0799 (4.8), 465.0681 (15.9), 313.0572 (26.8), 253.0363 (0.7), 193.0139 (1.9), 169.0132 (70.2), 151.0027 (1.8), 125.0230 (57.1), 107.0124 (6.7)	4.31	2.304	
64.	tellimagradin II 1	C_41_H_30_O_26_	937.0953	937.0970 (100), 785.0782 (0.3), 300.9991 (85.3), 275.0199 (18.1), 257.0094 (4.0), 249.0204 (12.32), 245.0090 (3.6), 229.0140 (7.1), 201.0188 (3.9), 169.0132 (13.8), 125.0230 (9.2), 107.0123 (1.9)	4.57	1.796	
65.	ellagic acid *O*-pentoside	C_19_H_14_O_12_	433.0412	433.0416 (100), 300.9990 (69.1), 271.9979 (2.5), 257.0092 (1.4), 245.0090 (0.6), 229.0139 (2.2), 201.0190 (1.5), 185.0237 (1.4), 145.0277 (0.4),	4.66	0.811	
66.	tellimagradin II 2	C_41_H_30_O_26_	937.0953	937.0970 (100), 785.0897 (3.8), 300.9992 (85.0), 275.0200 (19.2), 257.0091 (4.3), 249.0403 (11.3), 245.0087 (3.6), 229.0141 (6.3), 217.0139 (1.6), 201.0186 (3.8), 169.0131 (14.7), 125.0230 (10.4), 107.0124 (1.4)	4.74	1.726	
67.	tetragalloyl-hexose 1	C_34_H_28_O_22_	787.0999	787.1011 (100). 617.0776 (6.3), 465.0681 (32.2), 313.0572 (13.5), 295.0458 (7.8), 193.0135 (4.0), 169.0131 (73.7), 151.0025 (1.8), 125.0230 (70.0), 107.0122 (8.9)	4.94	1.518	[23]
68.	ellagic acid^a^	C_14_H_6_O_8_	300.9991	300.9990 (100), 257.0086 (1.6), 229.0143 (3.2), 217.0139 (0.9), 201.0182 (3.2), 185.0235 (2.3), 173.0235 (2.9), 145.0281 (3.1), 129.0332 (0.9), 117.0331 (1.0)	5.01	−0.101	[10]
69.	tetragalloyl-hexose 2	C_34_H_28_O_22_	787.0999	787.1019 (81.1). 617.0794 (57.9), 465.0681 (24.1), 313.0571 (10.1), 295.0459 (9.5), 193.0135 (4.2), 169.0123 (100), 151.0023 (3.0), 125.0230 (84.3), 107.0124 (10.4)	5.05	2.521	
**Flavonoids**							
70.	procyanidin dimer	C_30_H_26_O_12_	577.1351	577.1367 (100), 425.0887 (80.2), 407.0778 (66.3), 289.0721 (68.6), 245.0816 (4.7), 203.0710 (6.1), 179.0341 (6.0), 137.0232 (17.0), 125.0231 (89.8)	2.92	2.617	
71.	catechin/epicatechin	C_15_H_14_O_6_	289.0718	289.0719 (100), 245.0816 (41.3), 203.0707 (15.9), 179.0341 (10.1), 137.0231 (11.4), 109.0280 (34.0)	3.12	0.583	
72.	myricetin 3-*O*-galloylhexoside	C_28_H_24_O_17_	631.0941	631.0953 (100), 479.0839 (27.6), 317.0300 (15.9), 316.0226 (51.7), 299.0195 (3.1), 287.0197 (12.4), 271.0247 (20.5), 259.0253 (4.4), 243.0295 (2.3), 178.9972 (4.0), 169.0130 (4.5), 151.0022 (5.6), 125.0229 (5.7), 107.0125 (3.7)	4.19	1.914	[10,15,23]
73.	kaempferol 7-O-deoxyhexosylhexoside 1	C_27_H_30_O_15_	593.1512	593.1525 (95.1), 447.0926 (8.6), 431.0983 (5.7), 285.0406 (30.5), 255.0298 (30.5), 227.0342 (4.0), 211.0396 (2.9), 151.0025 (1.3), 107.0120 (0.5)	4.27	2.254	
74.	patuletin 3-*O*-dihexoside	C_28_H_32_O_18_	655.1516	655.1529 (100), 331.0439 (14.0), 330.0386 (73.2), 315.0151 (44.7), 287.0201 (16.2), 259.0246 (2.1), 243.0289 (3.9), 231.0300 (4.9), 215.0349 (5.6), 187.0393 (2.8), 165.9897 (4.3)	4.37	1.989	
75.	myricetin 3-*O*-hexoside1	C_21_H_20_O_13_	479.0831	479.0830 (100), 316.0226 (93.8), 317.0292 (20.8), 287.0199 (17.6), 271.0249 (26.7), 259.0247 (6.4), 243.0292 (3.6), 227.0351 (1.0), 178.9974 (3.4), 151.0025 (4.8), 107.0124 (1.6)	4.48	−0.217	[5,10,15,16,23]
76.	myricetin *O*-hexuronide	C_21_H_18_O_14_	493.0624	493.0634 (85.1), 317.0305 (100), 299.0199 (2.7), 271.0248 (3.9), 243.0297 (1.9), 227.0344 (1.3), 199.0391 (1.0), 178.9977 (15.6), 151.0025 (25.1), 137.0231 (16.8), 107.0124 (8.7)	4.51	1.423	[5,16]
77.	myricetin 3-*O*-hexoside 2	C_21_H_20_O_13_	479.0831	479.0836 (100), 317.0288 (17.9), 316.0226 (90.5), 287.0200 (17.1), 271.0248 (27.4), 259.0244 (7.0), 243.0291 (4.0), 227.0342 (0.9), 178.9975 (3.4), 151.0023 (3.9), 107.0121 (1.8)	4.57	0.994	[10]
78.	6-hydroxykaempferol methyl ether *O*-dihexoside	C_28_H_32_O_17_	639.1567	639.1581 (100), 459.0948 (0.7), 315.0499 (15.3), 314.0436 (64.2), 299.0199 (49.5), 271.0250 (25.7), 243.0298 (5.4), 227.0351 (3.4), 215.0343 (10.8), 199.0396 (2.4), 183.0445 (2.3), 165.9896 (6.6), 164.9817 (4.2), 136.9872 (0.7), 133.0278 (3.5), 109.9994 (3.0)	4.77	2.233	
79.	quercetin 3-*O*-galloylhexoside 1	C_28_H_24_O_16_	615.0992	615.1002 (100), 463.0890 (30.7), 301.0354 (30.4), 300.0277 (49.7), 271.0247 (31.7), 255.0299 (14.9), 243.0294 (5.2), 178.9977 (2.3), 169.0134 (9.6), 151.0026 (6.0), 125.0231 (11.8), 121.0283 (1.2), 107.0123 (4.4)	4.82	1.727	[5,10,16,23]
80.	kaempferol *O*-dihexoside	C_27_H_30_O_16_	609.1461	609.1473 (100), 429.0834 (2.2), 285.0395 (26.8), 284.0328 (82.9), 255.0298 (47.9), 227.0346 (32.2), 211.0397 (1.7), 178.9974 (1.6), 163.0030 (0.3), 151.0025 (2.8), 107.0121 (1.7)	4.85	2.023	
81.	quercetin galloylhexoside 2	C_28_H_24_O_16_	615.0992	615.1003 (100), 463.0889 (29.2), 301.0353 (30.4), 300.0274 (37.9), 255.0298 (14.1), 243.0298 (5.2), 178.9977 (2.8), 169.0130 (6.6), 151.0024 (7.2), 125.0231 (7.5), 121.0280 (1.3), 107.0123 (4.3)	4.96	1.825	[5,10]
82.	myricetin 3-*O*-deoxyhexoside	C_21_H_20_O_12_	463.0882	463.0888 (91.6), 317.0291 (26.3), 316.0227 (100), 287.0202 (17.7), 271.0248 (27.0), 259.0248 (5.4), 243.0293 (3.7), 227.0344 (0.6), 178.9974 (4.2)	5.11	1.384	[5,15]
83.	6-hydroxykaempferol methyl ether O-deoxyhexosylhexoside	C_28_H_32_O_16_	623.1618	623.1630 (100), 315.0493 (9.1), 314.0436 (67.1), 299.0200 (51.3), 271.0248 (26.7), 255.0299 (3.9), 243.0294 (5.2), 227.0346 (3.6), 215.0345 (11.8), 165.9897 (7.6), 164.9811 (4.9), 136.9867 (1.8), 133.0284 (4.8)	5.14	1.929	
84.	Isoquercitrin ^a^	C_21_H_20_O_12_	463.0885	463.0888 (100), 301.0349 (29.8), 300.0277 (63.8), 271.0249 (36.6), 255.0297 (14.9), 243.0296 (9.4), 227.0344 (2.6), 215.0343 (0.5), 199.0392 (0.9), 178.9978 (2.6), 163.0025 (1.8), 151.0025 (5.7), 121.0278 (1.0), 107.0124 (2.2)	5.19	1.319	[15,16,23];
85.	quercetin *O*-hexuronide	C_21_H_18_O_13_	477.0675	477.0682 (62.4), 301.0356 (100), 283.0245 (1.8), 255.0296 (3.6), 245.0450 (2.6), 227.0354 (1.3), 211.0396 (1.8), 178.9978 (9.7), 163.0022 (2.6), 151.0024 (21.7), 121.0281 (6.3), 107.0123 (8.2)	5.22	1.543	[5,10,15,16]
86.	hyperoside ^a^	C_21_H_20_O_12_	463.0885	463.0891 (100), 301.0351 (40.3), 300.0278 (71.9), 271.0249 (32.3), 255.0298 (13.9), 243.0296 (8.2), 227.0340 (2.7), 215.0343 (0.5), 178.9979 (3.3), 151.0025 (4.9), 121.0278 (1.5), 107.0121 (1.8)	5.29	1.837	[5,10,15,16]
87.	kaempferol-galloylhexoside 1	C_28_H_24_O_15_	599.1042	599.1055 (100), 447.0935 (2.9), 285.0401 (12.6), 284.0327 (12.0), 255.0296 (9.3), 241.0350 (6.6), 227.0350 (5.2), 211.0243 (1.9), 169.0133 (37.2), 151.0027 (4.2), 125.0230 (26.5), 107.0122 (5.6)	5.29	2.148	[10,16,23]
88.	quercetin *O*-pentoside	C_20_H_18_O_11_	433.0776	433.0781 (100), 301.0348 (23.1), 300.0277 (95.0), 271.0248 (49.6), 255.0295 (20.3), 243.0300 (10.6), 227.0353 (3.1), 178.9979 (2.1), 151.0023 (6.1), 107.0119 (2.6)	5.62	0.982	[5,10,15,16,23]
89.	kaempferol 3-*O*-glucoside ^a^	C_21_H_20_O_11_	447.0934	447.0945 (100), 285.0396 (15.6), 284.0328 (53.2), 255.0297 (38.2), 227.0345 (41.5), 211.0402 (1.5), 151.0029 (1.9), 107.0120 (0.5)	5.65	2.607	[16]
90.	kaempferol-galloylhexoside 2	C_28_H_24_O_15_	599.1042	599.1055 (100), 447.0931 (5.2), 285.0403 (17.4), 284.0326 (15.7), 255.0301 (11.9), 241.0349 (4.3), 227.0348 (7.0), 211.0230 (0.7), 169.0132 (31.6), 151.0026 (3.5), 125.0230 (24.5), 107.0124 (6.9)	5.67	2.148	[16]
91.	myricetin 3-*O*-caffeoylhexoside 1	C_22_H_22_O_12_	641.1160	641.1160 (100), 479.0840 (37.8), 317.0294 (23.6), 316.0227 (78.4), 287.0198 (17.6), 271.0249 (30.0), 259.0243 (2.9), 243.0291 (2.9), 178.9974 (4.6), 151.0022 (4.6), 107.0120 (1.1)	5.71	1.813	[16]
92.	kaempferol *O*-hexuronide	C_21_H_18_O_12_	461.0725	461.0732 (38.4), 285.0405 (100), 257.0450 (4.5), 229.0501 (6.6), 211.0393 (1.3), 175.0236 (5.0), 151.0021 (1.2), 107.01221 (2.1)	5.83	1.368	[5,10,15,16]
93.	myricetin 3-*O*-caffeoylhexoside2	C_22_H_22_O_12_	641.1160	641.1160 (100), 479.0840 (29.0), 317.0297 (27.7), 316.0227 (70.7), 287.0200 (16.8), 271.0249 (27.3), 259.0245 (4.9), 243.0294 (3.3), 178.9978 (5.1), 151.0024 (6.7), 107.0122 (2.2)	5.85	1.906	
94.	quercetin 3-*O*-deoxyhexoside	C_21_H_20_O_11_	447.0933	447.0940 (100), 301.0352 (71.7), 300.0279 (78.9), 271.0252 (31.7), 255.0297 (17.6), 243.0299 (7.3), 227.0351 (2.3), 178.9972 (4.4), 151.0023 (10.3), 121.0284 (2.9), 107.0123 (4.0)	5.90	1.645	[5,10,15,23]
95.	isorhamnetin 3-*O*-glucoside ^a^	C_22_H_22_O_12_	477.1038	477.1052 (100), 315.0497 (11.9), 314.0436 (54.4), 301.0359 (7.3), 271.0251 (20.5), 257.0454 (4.8), 243.0296 (22.2), 227.0351 (3.6), 199.0396 (3.1), 151.0024 (3.9), 107.0126 (0.7)	6.02	2.831	
96.	naringenin *O*-hexoside	C_21_H_22_O_10_	433.1140	433.1165 (4.5), 271.0613 (100), 227.0711 (0.2), 193.0138 (0.9), 151.0023 (21.0), 119.0487 (22.3), 107.0122 (2.6)	6.06	5.703	
97.	isorhamnetin O-hexuronide	C_22_H_20_O_13_	491.0831	491.0838 (49.3), 315.0514 (100), 300.0279 (25.8), 287.0570 (0.9), 271.0249 (26.8), 255.0298 (10.9), 243.0296 (7.7), 227.0340 (1.7), 215.0358 (0.4), 199.0385 (0.4), 175.0237 (5.6), 151.0025 (3.1), 107.0122 (1.3)	6.08	1.397	
98.	kaempferol 3-*O*-pentoside	C_20_H_18_O_10_	417.0827	417.0833 (100), 285.0393 (16.4), 284.0328 (63.3), 255.0298 (41.5), 227.0345 (38.1), 211.0391 (1.0), 151.0031 (0.8)	6.08	0.530	[15,16]
99.	kaempferol *O*-deoxyhexosylhexoside 2	C_27_H_30_O_15_	593.1512	593.1522 (77.4), 285.0406 (100), 229.0503 (11.8), 211.0393 (2.6), 163.0022 (1.9), 151.0022 (0.3), 135.0076 (1.3), 107.0124 (1.9)	6.32	1.697	
100.	myricetin *O*-*p*-coumaroylhexoside	C_30_H_26_O_15_	625.1199	625.1210 (85.5), 479.0835 (13.4), 317.0294 (18.9), 316.0227 (100), 287.0200 (15.4), 271.0250 (26.7), 259.0248 (6.3), 243.0301 (3.5), 178.9975 (4.3), 151.0026 (4.0), 107.0123 (1.8)	6.59	1.819	
101.	kaempferol *O*-deoxyhexoside	C_21_H_20_O_10_	431.0984	431.0988 (100), 285.0405 (78.8), 255.0299 (35.6), 227.0346 (30.6), 211.0390 (1.6), 163.0029 (0.9), 151.0012 (0.5), 135.0075 (0.5), 107.0124 (0.9)	6.59	0.380	[5,10,15,16,23]
102.	myricetin 3-O-feruloylhexoside	C_31_H_28_O_16_	655.1305	655.1322 (92.2), 479.0842 (8.7), 317.0290 (18.1), 316.0226 (100), 287.0200 (18.4), 271.0250 (26.1), 259.0249 (5.9), 243.0291 (4.7), 178.9978 (3.0), 151.0023 (4.5), 107.0122 (1.7)	6.74	2.659	
103.	kaempferol 7-*O*-caffeoylhexoside	C_30_H_26_O_14_	609.1250	609.1262 (100), 447.0934 (3.1), 285.0407 (75.3), 284.0331 (16.1), 255.0297 (14.6), 227.0345 (11.0), 211.0402 (1.0), 179.0340 (12.9), 161.0233 (37.6), 135.0438 (18.8), 133.0282 (13.6)	6.81	1.940	
104.	quercetin *O*-coumaroylhexoside 1	C_30_H_26_O_14_	609.1250	609.1260 (100), 463.0891 (22.7), 301.0353 (37.7), 300.0278 (59.7), 271.0250 (33.4), 255.0296 (15.6), 243.0297 (7.1), 227.0347 (3.0), 211.0397 (1.2), 178.9977 (2.6), 151.0025 (6.4), 121.0280 (1.3), 107.0122 (2.6)	7.05	0.643	[15,23]
105.	quercetin *O*-coumaroylhexoside 2	C_30_H_26_O_14_	609.1250	609.1260 (100), 463.0887 (30.0), 301.0352 (41.2), 300.0277 (78.9), 271.0250 (38.0), 255.0298 (16.6), 243.0295 (8.9), 227.0345 (2.5), 211.0398 (1.8), 178.9977 (2.7), 163.0031 (0.8), 151.0023 (6.6), 121.0281 (0.7), 107.0121 (2.1)	7.17	1.743	[16]
106.	quercetin 3-*O*-feruloylhexoside 1	C_31_H_28_O_15_	639.1355	639.1367 (100), 463.0887 (16.2), 301.0352 (37.7), 300.0277 (73.2), 271.0250 (36.0), 255.0297 (17.4), 243.0297 (11.1), 227.0351 (2.6), 178.9978 (3.4), 175.0393 (0.9), 151.0026 (8.0), 121.0277 (0.7), 107.0123 (2.8)	7.20	1.841	[16]
107.	quercetin 3-*O*-feruloylhexoside 2	C_31_H_28_O_15_	639.1355	639.1367 (100), 463.0887 (15.1), 301.0351 (31.9), 300.0278 (72.4), 271.0250 (37.3), 255.0300 (15.5), 243.0295 (9.3), 227.0344 (2.7), 199.0401 (0.6), 193.0502 (0.5), 178.9977 (2.7), 175.0398 (0.5), 161.0231 (1.3), 151.0025 (6.9), 121.0280 (1.4), 107.0121 (3.0)	7.32	1.841	
108.	quercetin *O*-coumaroylhexoside 3	C_30_H_26_O_14_	609.1250	609.1260 (100), 463.0888 (22.8), 301.0352 (38.1), 300.0278 (77.5), 271.0250 (40.5), 255.0298 (18.5), 243.0295 (10.3), 227.0344 (3.1), 211.0396 (1.2), 178.9980 (2.8), 163.0027 (1.6), 151.0025 (7.4), 121.0281 (1.3), 107.0125 (2.9)	7.54	1.743	
109.	kaempferol *O*-coumaroylhexoside 1	C_30_H_26_O_13_	593.1301	593.1313 (100), 447.0948 (4.0), 285.0403 (57.7), 284.0328 (68.0), 255.0298 (41.3), 227.0346 (28.0), 211.0396 (2.3), 163.0389 (1.1), 151.0024 (3.1), 145.0283 (11.5), 135.0075 (1.0), 119.0488 (2.7), 107.0125 (2.3)	7.58	2.168	[16]
110.	quercetin ^a^	C_15_H_10_O_7_	301.0354	301.0357 (100), 273.0410 (3.2), 257.0463 (0.6), 229.0505 (1.2), 211.0401 (0.4), 178.9978 (22.3), 151.0025 (44.3), 121.0281 (14.5), 107.0124 (15.4)	7.62		[17,23]
111.	kaempferol *O*-coumaroylhexoside 2	C_30_H_26_O_13_	593.1301	593.1313 (100), 447.0934 (2.0), 285.0403 (5.2), 284.0328 (52.4), 255.0298 (33.5), 227.0345 (24.5), 211.0395 (1.0), 163.0388 (2.5), 151.0024 (3.9), 145.0282 (8.0), 119.0488 (2.6), 107.0123 (2.0)	7.68	2.084	[16]
112.	kaempferol O-feruloylhexoside 1	C_31_H_28_O_14_	623.1406	623.1421 (100), 447.0935 (1.7), 323.0779 (2.7), 285.0401 (35.5), 284.0328 (63.0), 255.0298 (39.3), 227.0346 (28.4), 211.0397 (2.0), 193.0503 (1.6), 175.0394 (4.3), 161.0233 (8.1)151.0025 (2.2), 134.0358 (1.6), 107.0124 (1.6)	7.71	2.345	
113.	kaempferol O-feruloylhexoside 2	C_31_H_28_O_14_	623.1406	623.1419 (100), 447.0924 (1.5), 323.0770 (3.7), 285.0403 (61.1), 284.0327 (55.0), 255.0297 (41.4), 227.0344 (28.1), 211.0395 (3.1), 193.0500 (1.9), 175.0388 (4.5), 161.0231 (5.5), 151.0025 (3.6), 134.0359 (1.5), 107.0126 (2.7)	7.82		
114.	6-hydroxykaempferol methyl ether	C_16_H_12_O_7_	315.0510	315.0515 (80.4), 300.0279 (100), 272.0326 (8.1), 255.0302 (4.2), 243.0307 (1.2), 227.0345 (1.6), 165.9896 (8.0), 139.0025 (4.4), 136.9865 (4.4),	8.82	1.441	
115.	naringenin ^a^	C_15_H_12_O_5_	271.0612	271.0612 (12.6), 151.0020 (12.5), 119.0496 (10.2)	8.58	0.468	
116.	kaempferol ^a^	C_15_H_9_O_7_	285.0406	285.0404 (100), 257.0457 (0.9), 239.0352 (1.2), 229.0499 (0.9), 211.0399 (1.0), 187.0390 (1.4), 151.0024 (1.5), 107.0125 (1.2)	8.83	−0.215	[17]
117.	quercetin *O*-cinnamoylhexoside 1	C_30_H_26_O_13_	593.1301	593.1313 (100), 301.0346 (23.2), 300.0278 (80.2), 271.0248 (41.4), 255.0295 (9.6), 243.0295 (14.3), 227.0342 (4.0), 178.9976 (2.7), 151.0024 (5.0), 121.0279 (0.6), 107.0124 (2.3)	8.85	2.067	
118.	quercetin *O*-cinnamoylhexoside 2	C_30_H_26_O_13_	593.1301	593.1313 (100), 301.0349 (27.3), 300.0278 (73.8), 271.0251 (36.5), 255.0300 (10.0), 243.0297 (10.9), 227.0341 (3.1), 199.0392 (0.6), 178.9971 (2.8), 151.0024 (5.3), 135.0074 (1.0), 121.0283 (0.7), 107.0124 (2.4)	9.12	2.084	
119.	isorhamnetin ^a^	C_16_H_12_O_7_	315.0510	315.0515 (100), 300.0279 (47.0), 271.0241 (3.6), 255.0312 (1.2), 227.0346 (1.1), 164.0103 (3.2), 151.0023 (8.3), 107.0122 (7.0)	9.10	1.441	
120.	kaempferol *O*-cinnamoylhexoside	C_30_H_26_O_12_	577.1351	577.1361 (100), 285.0396 (17.2), 284.0328 (71.6), 255.0298 (49.7), 227.0345 (8.1), 211.0393 (1.1), 151.0024 (1.2),	9.50	−0.310	
121.	myricetin ^a^	C_15_H_10_O_8_	In (+)ESI-MS/MS 319.0448	319.0440 (100), 273.0383 (2.6), 245.0440 (4.4), 217.0491 (5.0), 165.0179 (2.5), 153.0180 (13.2)	9.49	−0.804	[15,17]

a—Identified by comparison with an authentic standard.

**Figure 2:**
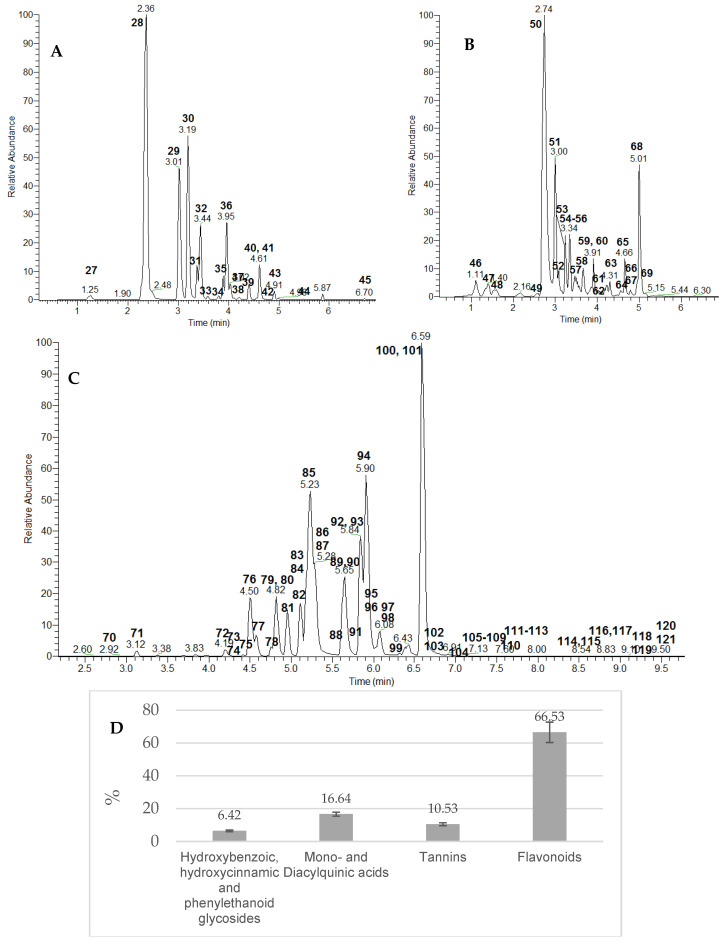
Extracted ion chromatograms of acylquinic acids (**A**), tannins (**B**), and flavonoids (**C**) in the *Epilobium angustifolium* extract; the percentage ratio of the main classes’ secondary metabolites (**D**). For peak numbering, see Table 1.

Sugar esters **1**, **10**, **14**, and **25** yielded prominent fragment ions resulting from the hexose cross ring cleavages and losses of 60 Da (^0,4^Hex), 90 Da (^0,3^Hex), and 120 Da (^0,2^Hex), as has been previously reported [20] (Figure S1). Methylgallate with [M-H]^−^ at *m*/*z* 183.029 was deduced from the typical loss of the methyl radical (•CH_3_) (−15 Da) at *m*/*z* 168.005 [24] (Table 1). The extracted ion chromatograms of hydroxybenzoic and hydroxycinnamic acids and derivatives showed that the *E. angustifolium* profile was dominated by gallic acid (**2**) (26.84%), methylgallate (**19**) (13.98%), coumaric acid-*O*-hexoside (**22**) (11.64%), and quinic acid (22) (11.52%) together with galloyl *O*-hexose (**1**) (10.27%). Overall, phenolic acids accounted for 6.42% of the assayed compounds (Figure 2D).

#### 2.1.2. Mono- and Diacylquinic Acids

Overall, 12 monoacylquinic and 7 diacylquinic acids were annotated/dereplicated on the base of preferential fragmentation reported elsewhere [24,25]. Thus, **27**, **28**, **29**, and **32** were assigned to 3-galloyl-, 3-caffeoyl (neochlorogenic), 3-*p*-coumaroyl-, and 3-feruloylquinic acid, respectively, while **30**, **35**, **36**, **39**, **41**, and **43** were ascribed as 5-substitidet quinic acid conjugates (Table 1, Figure 2A). Within the subclass of galloyl-caffeoylquinic acids at *m*/*z* 505.100 [M-H]^−^, four isomers, **33**, **37**, **38**, and **40**, were annotated. Compound **33** (consistent with molecular formula C_23_H_21_O_13_) gave a prominent ion at *m*/*z* 353.088 [M-H-galloyl]- and a base peak at *m*/*z* 191.055 [quinic acid-H]^−^, along with the abundant ions at *m*/*z* 179.034 [caffeic acid-H]^−^ and 135.044 [caffeic acid-H-CO_2_]^−^, as was seen for 3-caffeoylquinic acid (Table 1). In line with the fragmentation pattern of the 1,3-diacylquinic acids subclass [24], **33** was assigned to 1-galloyl-3 caffeoylquinic acid. Compound **37** afforded characteristic ions at *m*/*z* 353.088 (5.7%) and 343.068 [M-H-caffeoyl]^−^ (15.2%), indicating a loss of a caffeoyl residue before the galloyl one. This assignment was also suggested by the abundant ion at *m*/*z* 169.012, as was registered in 3-galloylquinic acid. Thus, **37** was ascribed as 1-caffeoyl-3-galloylquinic acid.

The base peak at *m*/*z* 191.055 suggested 1,5- (**38**) and 3,5-diacylquinic acid (**40**), supported by the relative abundances of the ions at *m*/*z* 179.034 and 135.044: 3.1% and 3.0% in 1-galloyl-5-caffeoylquinic acid, and 56.8% and 54.2% in 3-caffeoyl-5-galloylquinic acid, respectively (Table 1; Figure S2). In the same manner, **44** (3-galloyl-5-*p*-coumaroylquinic acid) produced a base peak at *m*/*z* 191.055, accompanied with low abundant peaks at *m*/*z* 163.040 (4.35%) and 119.049 (1.2%), as was seen in 5-*p*-coumaroylquinic acid (Table 1).

Among acylquinic acids, being present at 16.64% of the studied compounds (Figure 2D), neochlorogenic acid (**28**) (35.11%), together with its isomer chlorogenic acid (**30**) (16.91%), and 3-*p*-coumaroylquinic acid (18.23%) appeared to be dominant for the *E. angustifolium* extract. Overall, neochlorogenic acid content in leaves was three times higher compared to the flowers, being present up to 5.78 mg/g dw and 1.82 mg/g dw, respectively [5].

#### 2.1.3. Gallotannins and Ellagitannins

Four compounds (**48, 49, 52**, and **56**) were assigned to digalloyl-hexose isomers. The MS/MS fragmentation pathway afforded indicative ions, resulting from the losses of galloyl residue at *m*/*z* 331.068 [M-H-C_7_H_4_O_4_]^−^ and gallic acid (GA) at *m*/*z* 313.057 [M-H-C_7_H_6_O_5_]^−^ along with abundant ions at *m*/*z* 169.013 [GA-H]^−^ and 125.023 [GA-H-CO_2_]^−^. Peaks **57**–**60** were tentatively identified as trigalloyl-hexose isomers, owing to their [M-H]^−^ at *m*/*z* 635.090 and typical MS/MS fragments at *m*/*z* 483.079 [M-H-galloyl]^−^, 313.057 [M-H-2galloyl-H_2_O]^−^, and 169.013 [M-H-2galloyl-Hex]^−^ (Table 1). Regarding **67** and **69**, four galloyl residues were deduced from the following transitions: 787.101→617.078 [M-H-GA]^−^→465.068 [M-H-2galloyl-H_2_O]^−^→313.057 [M-H-3galloyl-H_2_O]^−^ (Table 1). Accordingly, the aforementioned compounds were annotated as tetragalloyl-hexose isomers [24]. Three isobars, **46, 47**, and **53**, shared the same [M-H]^−^at *m*/*z* 633.075; the abundant fragment ion at *m*/*z* 300.999 (ellagic acid, EA) indicated the loss of a galloyl-hexose moiety (332 Da, C_13_H_16_O_10_) (Table 1). This assumption was in line with the presence of a series of indicative ions for EA, including 275.020 257.009 [EA-H-CO_2_]^−^, 229.014 [EA-H-CO-CO_2_]^−^, 185.023 [EA-H-2CO-2CO_2_]^−^, and 145.029 [EA-H-4CO-CO_2_]^−^(Table 1) [21]. Consequently, the abovementioned compounds were ascribed as galloyl-HHDP-hexose isomers. In the same way, tellimagradin I isomers **51** and **58** at *m*/*z* 785.086(7) [M-H]^−^ were discernable by the characteristic loss of 484.087 Da (C_20_H_20_O_14_) and the prominent ion at *m*/*z* 300.999 (Table 1). Compounds **64** and **66** at *m*/*z* 937.0970 differ from the aforementioned compounds with 152 Da (galloyl residue) and were consistent with tellimagradin II [26].

The MS/MS spectrum of compound **55** ([M-H]^−^ at *m*/*z* 291.045, C_13_H_7_O_8_) was acquired. The fragmentation pattern was delineated by the base peak at *m*/*z* 247.025 [M-H-CO_2_]^−^ and the following transitions at *m*/*z* 219.030: [M-H-CO-CO_2_]^−^→191.034 [M-H-2CO-CO_2_]^−^→173.023[M-H-2CO-CO_2_-H_2_O]^−^→145.028 [M-H-3CO-CO_2_-H_2_O]^−^, as has been seen in brevifolin carboxylic acid [24].

Compound **50** was ascribed as the macrocyclic ellagitannin dimer oenothein B, previously reported from *Epilobium* species [3]. The dimer consists of two 784 Da units (tellimagradin I) and has a molecular mass of 1568.1518 Da (C_68_H_48_O_44_). In the MS/MS spectrum, **50** afforded prominent ions [M-2H]^2−^ at *m*/*z* 783.070 (consistent with C_68_H_46_O_44_) and at *m*/*z* 300.9992 [EA-H]^−^, resulting from the loss of C_20_H_20_O_14_ (484.087 Da = 152 + 152 + 162 + 18 Da). It should be noted that in the extracted ion chromatogram, Oenonthein B showed two isomers—the minor one could be seen eluting after the main isomer at t_R_ 3.74 (both isomers had identical MS/MS patterns) (Figure S3). In this respect, Karonen et al. [27] reported that in HPLC-MS analysis, the purified oenothein B shows a mixture of two isomers in a 95:5 ratio.

Compounds **57** and **59** were assigned to oenothein A isomers. The ellagitannin trimer oenothein A has an additional monomer (784 Da) compared to oenothein B and a molecular mass of 2352.228 Da, consistent with a molecular formula C_102_H_72_O_66_. Both isomers were tentatively identified by the double-charged ions at *m*/*z* 1175.609 [M-2H]^2−^. They gave indicative fragment ions at *m*/*z* 785.085 [tellimagradin I-H]^−^, 633.075 [galloyl-HHDP-hexose]^−^, 427.031 [galloyl-HHDP-hexose-4H_2_O]^−^, and 399.036 [galloyl-HHDP-hexose-4H_2_O-CO]^−^, along with the abundant fragment ion at *m*/*z* 300.9992 ([EA-H]^−^). The trimeric Oenothein A showed a major isomer at t_R_ 3.44 and a minor one, in contrast to the study of Karonen et al. [27], where two major isomers in a 50:50 ratio were recorded. Among gallo- and ellagitannins, reaching levels of up to 10.53%, oenothein B isomer 1 (**50**) (53.29%), followed by ellagic acid acid (**68**) (14.12%), galloyl-HHDP-hexose isomer 3 (**53**) (4.62%), and ellagic acid O-pentoside (**65**) (3.61%), were found to be dominant for *E. angustifolium* extract (Figure 2B).

It is well known that the polyphenol profiling of fireweed is dominated by oenothein B [5,10,16,17]. In the study of Baert et al. [5] on the intra-organ distribution of ellagitannins, both oenothein B and A have been found in significant levels, reaching 148.28 mg/g dw and 52.14 mg/g dw, respectively, in the inflorescence apex of the species. It is noteworthy that the oenothein B was twice as much as that determined in the leaves. Moreover, oenothein B was more abundant in the flowers (up to 102.27 mg/g dw) in comparison with the leaves.

#### 2.1.4. Flavonoids

Procyanidin dimer (**70**) at *m*/*z* 577.137 and catechin/epicatechin (**71**) at *m*/*z* 289.072 were deduced from the indicative fragment ions at *m*/*z* 245.082, 203.071 and 137.023 (Table 1) [26].

A variety of acylhexosyl flavonols were tentatively identified, including kaempferol, quercetin, and myricetin glycosides (Figure 2C). The approach for flavonol annotation has been delineated elsewhere [20]. Compounds **72, 79, 81, 87**, and **90** shared similar fragmentation patterns, affording indicative fragment ions at *m*/*z* 447.093 (**87** and **90**), 463.089 (**79** and **81**), and 479.084 (**72**), resulting from the loss of a galloyl moiety (152 Da, C_7_H_4_O_4_) from kaempferol, quercetin, and myricetin glycosides, respectively (Table 1). Moreover, the galloyl residue was evidenced by the fragment ions at *m*/*z* 169.013, 151.002, and 125.023. The glycosylation position at C-3 was discernable by the low abundant Y_0_^−^ aglycone ions compared with the radical aglycone ions [Y_0_-H]^−●^, as typical MS/MS fragmentation behavior of flavonoid 3-*O*-glycosides [24]. Consequently, **72** was ascribed as myricetin 3-*O*-galloylhexoside, while **79** and **81** were assigned to quercetin 3-*O*-galloylhexosides, and **87** and **90** to kaempferol 3-*O*-galloylhexosides (Figure S4). In the same manner, **91**/**93** and **103** were annotated as caffeoyl esters of myricetin- and kaempferol-hexoside, respectively. The caffeoyl residue was deduced from the losses of 162 Da (C_9_H_6_O_3_) and 324 Da (C_15_H_16_O_8_), supported by the prominent fragments at *m*/*z* 179.034, 161.023, and 135.044 (Table 1). Coumaroyl esters of myricetin- (**100**), quercetin- (**104**, **105**, and **108**), and kaempferol-hexoside (**109** and **111**) were discernable by the transitions—[M-H]^−^→(Y_0_-H)^−●^—resulting from the losses of 309 Da (C_15_H_17_O_7_). Accordingly, feruloylhexosides **102**, **106**, **107**, **112**, and **113** were witnessed by the indicative losses of 176 Da (C_10_H_8_O_3_) and 339 Da (C_16_H_19_O_8_) from the corresponding precursor ions. Two quercetin-cinnamoylhexosides (**117** and **118**) and kaempferol-cinnamoylhexoside (**120**) were evidenced on the basis of the loss of 293 Da ([M-H]^−^→(Y_0_-H)^−●^).

Compounds **76, 85, 92**, and **97** were closely related with the same fragmentation pathway, giving ([M-H-HexA]^−^, suggesting hexuronides of myricetin, quercetin, kaempferol, and isorhamnetin, respectively. In the same manner, deoxyhexosylhexoside of kaempferol (**73, 99**) and 6-hydroxykaempferol methyl ether (**83**) were tentatively identified (Table 1).

In (-) ESI-MS/MS compounds, **74** and **78** afforded prominent fragment ions at *m*/*z* 331.044 and 315.050, respectively, indicating the presence of a dihexosyl moiety. The deprotonated aglycone molecules underwent the loss of a methyl radical at *m*/*z* 315.015 and 299.020, respectively. Low abundant RDA ions at *m*/*z* 165.990 (^1,3^A^−^-^●^CH_3_) (**70** and **74**), 164.982 (^1,3^A^−^-CH_4_), and 136.987 (^1,3^A^−^-CH_4_-CO) (**78**) a suggested 6-methoxylated A-ring of the flavonoid aglycone. Accordingly, the aforementioned compounds were annotated as patuletin 3-*O*-dihexoside (**74**) and 6-hydroxykaempferol methyl ether *O*-dihexoside (**78**).

Based on the comparison with the fragmentation patterns and retention times of reference standards, **84**, **86**, **89**, **95**, **110**, **116**, **119**, and **121** were unambiguously identified as isoquercitrin, hyperoside, kaempferol 3-*O*-glucoside, isorhamnetin 3-*O*-glucoside, quercetin, kaempferol, isorhamnetin, and myricetin. Overall, kaempferol *O*-deoxyhexoside (**101**) (23.19%), quercetin 3-*O*-deoxyhexoside (**94**) (14.73%), and quercetin *O*-hexuronide (**85**) (13.84%) were found to be the predominant flavonoids, followed by kaempferol *O*-hexuronide (**92**) (7.10%), 6-hydroxykaempferol methyl ether *O*-dihexoside (**78**) (3.99%), and isoquercitrin (**84**) (3.68%) (Figure 2C).

According to the comparison of the relative abundance and the percentage ratio of all annotated compounds, flavonoids were found to be the main classes’ secondary metabolites found in *E. angustifolium* aerial parts (66.53% ± 6.23%) (Figure 2D).

These results were consistent with the earlier reports on the *E. angustifolium* distribution of the main polyphenols, where the highest value of the sum of hyperoside, quercetin 3-*O*-glucuronide, and quercitrin was determined with an intra-population mean content of 9.4 mg/g dw [3]. Quercetin 3-*O*-glucuronide has been previously reported as the major compound in *E. angustifolium* [5,11]. Baert et al. [5] reported up to 6.19 mg/g dw in the leaves, while significantly lower values were found in the flowers (up to 2.83 mg/g dw). Both, quercetin- and kaempferol-3-*O*-glucuronide were identified as chemotaxonomic markers of the species; the latter was present in similar amounts in leaves and flowers [5,11]. It is noteworthy that in our study, the flavonoid profile was dominated by kaempferol 3-*O*-deoxyhexoside. The highest levels of this compound have been recorded in fireweed flowers (up to 6.76 mg/g dw), while it was completely absent from the leaves [5]. In Baert’s study, hyperoside showed similar levels in the majority of the studied populations, being present from 0.15 to 0.38 mg/g dw (up to 1.48 mg/g dw in the leaves). Myricetin 3-*O*-glucoside and myricetin 3-*O*-glucuronide exhibited the greatest inter-population variability, ranging from below the limit of quantitation to the average content of 2.31 mg/g dw. In the same study, quercetin 3-*O*-(6-galloyl)-galactoside reached up to 0.56 mg/g dw in the flowers, while it was found in a very low concentration in the leaves. Furthermore, kaempferol-, quercetin-, and myricetin 3-*O*-glucuronide were unique to the flowers, accounting for 70–85% of flower flavonoid content.

### 2.2. Total Phenolic and Flavonoid Content

Phenolic compounds are considered effective therapeutic aids for preparing health-promoting applications. In this sense, determining the total phenolic content provides a first insight into the potential of the plants [28]. In the present study, the total phenolic and flavonoid content of the methanol aqueous extract of *E. angustifolium* was determined using spectrophotometric assays (Table 2). Our results revealed a higher concentration of polyphenols (85.04 ± 0.18 mg GAE/g) and lower level of flavonoids (27.71 ± 0.74 mg QE/g) in comparison with the previous records [29]. Moreover, Monschein et al. [30] have found that the total content of phenolic compounds in *E. angustifolium* showed no correlation with the altitudinal level. The extracts contained phenolics between 97.6 and 135.2 mg/g (800 m: 113.7 ± 15.71 mg/g; 1000 m: 123.0 ± 11.23 mg/g; 1500 m: 114.8 ± 1.60 mg/g). [30]. In addition, Szwajgier et al. [17] reported that the concentration of total polyphenols and flavonoids in the freeze-dried infusion was 407.02 ± 7.10 mg GAE/g dry material and 53.04 ± 1.24 mg QE/g, respectively. However, oenothein B, the compound unique to fireweed, was not present, due to the different sample preparation and extraction solvent [17]. In another study by Ak et al. [31], the total phenolic content and flavonoid content in *E. hirsutum* extracts was determined as 43.52–254.55 mg GAE/g and 10.87–87.66 mg rutin equivalent (RE)/g. In recent times, spectrophotometric methods have faced criticism due to their lack of specificity, as reagents can interact with both phenolic and non-phenolic compounds, potentially yielding inaccurate results [32]. To ensure accuracy, spectrophotometric findings should be confirmed through chromatographic methods like LC-MS/MS or NMR.

### 2.3. Antioxidant Capacity

Antioxidants are effective defenses against attacks by free radicals, preventing the progression of serious chronic and degenerative diseases such as cancer, diabetes, and cardiovascular diseases. In the current study, the antioxidant properties of the methanol-aqueous extract of *E. angustifolium* were investigated using different methods. The results are presented in Table 2.

DPPH and ABTS are the most commonly used free radicals to evaluate scavenging ability. In particular, through the transfer of hydrogen from antioxidants, the radicals can be intercepted, and the changes can be determined using spectrophotometric measurements. CUPRAC and FRAP assays are used to evaluate the electron donation ability of antioxidants, referred to as their reducing ability. A high reducing ability reflects a high antioxidant effect. In addition, the phosphomolybdenum test involves the conversion of Mo(VI) to Mo(V). The ability to chelate metals is associated with controlling the production of hydroxyl radicals in the Fenton reaction. As can be seen in Table 2, the tested extract showed significant radical scavenging and reduction effects as well as metal chelation. The radical scavenging activity of *E. angustifolium* has been reported previously [11,14,33]. Hevesy et al. [11] evaluated ABTS radical scavenging activity of five *Epilobium* species, with EC_50_ ranging between 1.71 and 3 μg/mL—the highest activity levels possessed by *E. parviflorum*. Deng et al. [14] reported that the *E. angustifolium* ethanol extract possessed antioxidant activities in DPPH and ABTS assays with EC_50_ at 25.53 ± 0.40 and 45.71 ± 1.34 μg/mL, respectively, and inhibited lipid peroxidation (%inhibition 26.74 ± 2.03). The highest radical scavenging ability in DPPH and ABTS assays was found for the ethylacetate extract in comparison with the petroleum ether extract. Owing to the high content of oenothein B in many Oenothera, *Epilobium,* and Eucalyptus species, its biological activity has been widely studied [22]. Numerous investigations demonstrated that oenothein-rich *Epilobium* sp. significantly reduced the production of reactive oxygen species (ROSs), which may hold significance for reducing the risk of diseases associated with active oxygen damage [12,22,34]. These effects were ascribed to the radical scavenging capacity of the ellagitannins, which are able to terminate free radical chain reactions of lipids, proteins, and DNA by self-oxidation [22].

In vivo studies showed that oenothein B exerts neuroprotective effects on the central nervous system, mitigates neuroinflammation in the brain, and enhances neuronal signaling pathways [34]. Indeed, oenothein had the highest radical scavenging activity among the other polyphenols (quercetin 3-O-glucuronide and myricetin 3-O-rhamnoside) in fireweed methanol extracts [12,33]. In fireweed herb, a correlation between radical scavenging activity and total phenolic content, notably oenothein B, has been evidenced [33]. It is noteworthy that the metabolites of various ellagitannins may have more pronounced antioxidant activity compared to the corresponding compounds [13].

### 2.4. Enzyme Inhibitory Activity

Enzymes are important targets in pharmaceutical therapies, and many treatment approaches focus on modulating enzymes. By inhibiting critical enzymes, symptoms of diseases such as diabetes, obesity, and Alzheimer’s can be treated. For example, suppressing amylase and glucosidase activities helps control blood sugar levels in diabetics following a high-carbohydrate diet [35]. Although synthetic enzyme inhibitors, including acarbose, galanthamine, and kojic acid, have been synthesized, their adverse side effects remain a challenge. Therefore, it is important to identify safer and more effective natural inhibitors [36]. The enzyme inhibitory activity of the studied extracts was determined against acetyl- and butyrylcholinesterase, tyrosinase, α-amylase, and α-glucosidaseand lipase (Table 2). The studied extract showed moderate acetylcholinesterase (2.05 mg GALAE/g) and butyrylcholinesterase (1.67 mg GALAE/g) inhibitory activity.

In addition, the extract displayed very high potential against the enzyme tyrosinase (61.94 ± 0.05 mg KAE/g) (Table 2). This enzyme plays a key role in the biosynthesis of melanin and is responsible for skin pigmentation. Increased melanin formation leads to skin diseases such as hyperpigmentation and skin spots, amongst others. Tyrosinase inhibitors are becoming increasingly important as hypopigmenting active ingredients in cosmetics and pharmaceuticals [37].

Regarding enzymes involved in carbohydrate and lipid metabolism, *E. angustifolium* revealed low α-amylase (0.44 mmol ACAE/g) and high α-glucosidase (3.48 mmol ACAE/g) and lipase inhibitory (8.03 mg OE/g) effects. The inhibition of these enzymes is known to be an important therapeutic strategy to control blood glucose levels in diabetic patients who have had a carbohydrate-rich diet and metabolic disorders. In the literature, some researchers have reported enzyme inhibitory effects of the members of the genus *Epilobium* [12,38,39,40]. For example, Ak et al. [31] reported that the AChE and BChE inhibitory activity was 2.69–4.48 mg GALAE/g and 1.11–4.72 mg GALAE/g for the extracts of the aerial parts of *E. hirsutum*, respectively. In addition, the amylase and glucosidase inhibitory effects were found to be 0.17–1.02 mmol ACAE/g and 1.57–1.62 mmol ACAE/g, respectively, in their study. In terms of insights into the structure–ability relationship based on Table 1, some identified compounds in the tested extracts may contribute to the observed enzyme inhibitory effects. For example, quercetin, gallic acid, caffeic acid, kaempferol, and myricetin have been already recognized as enzyme inhibitors in previous reports [41,42,43,44,45,46,47,48,49]. In this sense, the tested *E. angustifolium* extract could be considered as a multifunctional bioactive agent from antioxidants to enzyme inhibitors, and, thus, the presented study could be valuable to provide an effective raw material in the pharmaceutical, nutraceutical, and cosmeceutical industries.

## 3. Materials and Methods

### 3.1. Plant Material

Fireweed is distributed in the Vitosha Mt. floristic region in Bulgaria, where the locality was chosen with the following criteria: (1) a representative subalpine region; (2) the species is common and extensive in the herbaceous community; (3) a lack of anthropogenic activity; (4) the diversity of higher plants in the Vitosha Mt. represents 41% of the total species for Bulgaria [50]. *E. angustifolium* aerial parts (Epilobii Herba) were collected at the locality “Platoto”, Vitosha Mt. (1906 m. a.s.l.), Bulgaria, during the full flowering stage in July 2023. The species taxonomic identity was confirmed by one of us (R. Gevrenova) according to www.worldforaonline.org. A voucher specimen was deposited at Herbarium Facultatis Pharmaceuticae Sophiensis, Medical University-Sofia, Bulgaria (Voucher specimen No. 11823). The plant material was dried at room temperature.

### 3.2. Sample Extraction

*E. angustifolium* aerial parts were powdered by a grinder (Rohnson, R-942, 220–240 V, 50/60 Hz, 200 W, Prague, Czech Republic). Powdered plant material (50 g) was extracted with 80% MeOH (1:20 *w*/*v*) by sonication (100 kHz, ultra-sound bath Biobase UC-20C) for 15 min (×2) at room temperature. The methanol was evaporated in vacuo (40 °C) and water residues were lyophilized (lyophilizer Biobase BK-FD10P; −65 °C) to yield 7.3 g of crude extract. Then, the lyophilized extracts were dissolved in 80% methanol (0.1 mg/mL), filtered through a 0.45 μm syringe filter (Polypure II, Alltech, Lokeren, Belgium), and an aliquot (2 mL) of each solution was subjected to UHPLC–HRMS analyses. The same extracts were used for in vitro antioxidant and enzymatic capacity tests.

### 3.3. Chemicals

Acetonitrile (hypergrade for LC–MS), formic acid (for LC–MS), and methanol (analytical grade) were provided from Chromasolv (Sofia, Bulgaria). The reference standards used for compound identification were obtained from Extrasynthese (Genay, France) (for gallic, protocatechuic, 4-hydroxybenzoic, 3-hydroxybenzoic, o- and p-coumaric, caffeic, and salicylic acids, myricetin, hyperoside, isoquercitrin, kaempferol 3-O-glucoside, isorhamnetin 3-O-glucoside, quercetin, isorhamnetin, and kaempferol) and Phytolab (Vesten-bergsgreuth, Bavaria, Germany) (ellagic acids and naringenin). A working solution containing 0.1 mg/mL of the assayed compounds was prepared from a stock solution in methanol containing 0.5 mg/mL.

The chemicals for antioxidant and enzyme inhibition assays were purchased from Sigma-Aldrich (Darmstadt, Germany). They were as follows: ABTS, DPPH, gallic acid, rutin, electric eel acetylcholinesterase (AChE) (type-VI-S, EC 3.1.1.7), horse serum butyrylcholinesterase (BChE) (EC 3.1.1.8), galantamine, acetylthiocholine iodide (ATChI), butyrylthiocholine chloride (BTChI) 5,5-dithio-bis(2-nitrobenzoic) acid (DTNB), tyrosinase (EC1.14.18.1, mushroom), glucosidase (EC. 3.2.1.20, from *Saccharomyces cerevisiae*), amylase (EC. 3.2.1.1, from porcine pancreas), sodium molybdate, sodium carbonate, Folin–Ciocalteu reagent, hydrochloric acid, sodium hydroxide, trolox, ethylenediaminetetraacetate (EDTA), neocuproine, cupric chloride, ammonium acetate, ferric chloride, 2,4,6-Tris(2-pyridyl)-s-triazine (TPTZ), ammonium molybdate, ferrozine, ferrous sulfate hexahydrate, kojic acid, and acarbose. All chemicals were of analytical grade.

### 3.4. UHPLC-HRMS

The UHPLC-HRMS analyses were performed as previously described [51] on a Q Exactive Plus mass spectrometer (ThermoFisher Scientific, Inc., Waltham, MA, USA) equipped with a heated electrospray ionization (HESI-II) probe (ThermoScientific). The equipment was operated in negative and positive ion modes within the *m*/*z* range of 150 to 1500. The chromatographic separation was achieved on a Kromasil Eter-nityXT C18 (1.8 µm, 2.1 × 100 mm) reversed-phase column, at 40 °C. The UHPLC analyses were run with a mobile phase consisting of 0.1% formic acid (A) and 0.1% formic acid in acetonitrile (B). The run time was 33 min and the flow rate was 0.3 mL/min. The used gradient elution program was as follows: 0–1 min, 0–5% B; 1–20 min, 5–30% B; 20–25 min, 30–50% B; 25–30 min, 50–70% B; 30–33 min, 70–95%; 33–34 min 95–5% B. The equilibration time was 4 min. The injection volume was 1 µL, and the flow rate was 300 µL/min. Data were processed by Xcalibur 4.2 (ThermoScientific, Waltham, MA, USA) instrument control/data handling software. MZmine 2 software was applied to the UHPLC–HRMS raw files of the studied *E. angustifolium* extract for the semi-quantitative analysis. Results are expressed as the % peak area of the compound to the total peak areas of the corresponding group secondary metabolites and all metabolites.

### 3.5. Assay for Total Phenolic and Flavonoid Contents

According to the methods specified by Zengin and Aktumsek [52], total phenolics and flavonoids were quantified. The extract was prepared at a concentration of 2 mg/mL. Gallic acid (GA) and rutin (R) were used as positive equivalents in the assays, and the results were reported as gallic acid equivalents (GAEs) and rutin equivalents. The values of the calibration curves are as follows: for the total phenolic content, absorbance = 0.268 [μg gallic acid] (R^2^: 0.9988, concentration range: 0–3 μg gallic acid); for total flavonoid content, absorbance = 0.1274 [μg rutin] + 0.0506 (R^2^: 0.9968, concentration range: 0–20 μg rutin).

### 3.6. Assays for In Vitro Antioxidant Capacity

According to the methods provided by Zengin et al. [53], antioxidant tests were executed. The extract was prepared at concentrations of 0.1–2 mg/mL. The DPPH, ABTS radical scavenging, CUPRAC, and FRAP results were expressed as milligrams of Trolox equivalents (TE) per gram of extract. The antioxidant potential determined by the phosphomolybdenum (PBD) assay was presented in millimoles of Trolox equivalents (TE) per gram of extract. Metal chelating activity (MCA) was calculated as milligrams of disodium edetate equivalents (EDTAE) per gram of extract. The experimental details are presented in the Supplemental Materials.

### 3.7. Inhibitory Effects Against Some Key Enzymes

Enzyme inhibition experiments on the samples were carried out following established protocols [53]. The extract was prepared at a concentration of 0.1–2 mg/mL. Amylase and glucosidase inhibition were expressed as acarbose equivalents (ACAE) per gram of extract, while acetylcholinesterase (AChE) and butyrylcholinesterase (BChE) inhibition were evaluated as milligrams of galanthamine equivalents (GALAE) per gram of extract. Tyrosinase inhibition was evaluated in milligrams of kojic acid equivalents (KAE) per gram of extract. Lipase inhibition was measured as the equivalent of orlistat (OE) per gram of extract. The experimental details are presented in the Supplemental Materials.

### 3.8. Statistical Analysis

The experiments for the evaluation of total phenolic and flavonoid contents and antioxidant and enzyme inhibitory capacity were performed in triplicate and the results were presented as the mean and standard deviation. GraphPad 9.1 was used to evaluate the obtained results.

## 4. Conclusions

Herein, an in-depth phytochemical profiling of the methanol-aqueous extract from *E. anfustifolium* aerial parts with Bulgarian provenance was performed by the means of liquid chromatography–Orbitrap high-resolution mass spectrometry. More than 120 secondary metabolites—notably, phenolic acids and their glycosides, acylquinic acids, gallotannins and ellagitannins, and flavonoids—were dereplicated/annotated. Forty-six compounds (11 phenolic acids, 10 acylquinic acids, 3 tannins, and 22 flavonoids) are reported for the first time in the species. Furthermore, links between the compounds having similar HRMS/MS fragmentation patterns were established, which greatly help the metabolite annotation of galloyl-caffeoylquinic acids, mono-, di-, and trigalloyl-hexose, galloyl, caffeoyl, feruloyl, and p-coumaroyl conjugates of flavonol hexosides. The percentage ratio of the main compound classes revealed the highest relative content of flavonoids (66.5 ± 6.23%), followed by mono- and diacylquinic acids (16.6 ± 1.2%) and tannins (10.53 ± 0.89%). Gallic and ellagic acids, methylgallate, neochlorogenic and chlorogenic acids, oenothein B, galloyl-HHDP-hexose, as well as deoxyhexosides of kaempferol and quercetin, along with a variety of acylated flavonol hexosoides, appeared to be characteristic for *E. anfustifolium* aerial parts. The strong antioxidant potential (DPPH^•^, ABTS^•+^, FRAP, and CUPRAC) could be assigned to dimeric and trimeric ellagitannins together with simple phenolics (gallic acid and methylgallate), and numerous caffeoyl, coumaroyl, and feruloyl conjugates. Indeed, the polyphenols are the main group in fireweed with an impact on the anti-cholinesterase and anti-tyrosinase activity. In addition to evoking an antioxidant response, *E. angustifolim* extract exhibited in vitro inhibitory activity towards key enzymes of melanin biosynthesis and carbohydrate and lipid metabolism, which generates further interest in the herbal drug Epilobii Herba as a potential therapeutic candidate for associated disorders.

## Figures and Tables

**Table 2:** Total bioactive compounds, antioxidant properties, and enzyme inhibitory properties of tested extract.

*Total bioactive compounds*	
Total phenolic content (mg GAE/g)	85.04 ± 0.18
Total flavonoid content (mg RE/g)	27.71 ± 0.74
*Antioxidant properties*	
DPPH scavenging ability (mg TE/g)	310.74 ± 11.09
ABTS scavenging ability (mg TE/g)	466.82 ± 23.60
CUPRAC (mg TE/g)	442.83 ± 12.27
FRAP (mg TE/g)	291.50 ± 4.32
Metal chelating (mg EDTAE/g)	48.20 ± 0.44
Phosphomolybdenum (mmol TE/g)	2.10 ± 0.09
*Enzyme inhibitory properties*	
AChE inhibition (mg GALAE/g)	2.05 ± 0.04
BChE inhibition (mg GALAE/g)	1.67 ± 0.07
Tyrosinase inhibition (mg KAE/g)	61.94 ± 0.05
Amylase inhibition (mmol ACAE/g)	0.44 ± 0.01
Glucosidase inhibition (mmol ACAE/g)	3.48 ± 0.08
Lipase inhibition (mg OE/g)	8.03 ± 0.11

Values are reported as mean ± SD of three parallel measurements. GAE: gallic acid equivalent; RE: rutin equivalent; TE: trolox equivalent; EDTAE: EDTA equivalent; GALAE: galanthamine equivalent; KAE: kojic acid equivalent; ACAE: acarbose equivalent; OE: orlistat equivalent.

## Data Availability

Data are contained within the article.

## Supplementary Materials

The following supporting information can be downloaded at: https://www.mdpi.com/article/10.3390/plants14030415/s1, Figure S1: (-) ESI-MS/MS spectrum of galloyl-(caffeoyl)-hexose (25); Figure S2: ESI-MS/MS spectrum of 1-galloyl-5-caffeoylquinic acid (38); Figure S3: (-) ESI-MS/MS spectrum of oenothein B (50); Figure S4: (-) ESI-MS/MS spectrum of quercetin 3-O-galloylhexoside (79); Determination of Antioxidant and Enzyme Inhibitory Effects.
