# Supplementary material for: Exploring the Phytochemical Profile and Biological Insights of Epilobium angustifolium L. Herb
Source: Plants (Basel). 2025 Jan 31;14(3):415. doi: 10.3390/plants14030415 (PMC11820171; doi:10.3390/plants14030415)
Supplement: Supplementary file 1 [file plants-14-00415-s001.zip › plants-3407257-supplementary.pdf]

Exploring the Phytochemical Profile and Biological Insight into *Epilobium angustifolium* L. Herb

Reneta Gevrenova [1]*, Gokhan Zengin [2], Gulsah Ozturk [2] and Dimitrina Zheleva-Dimitrova [1]

[1] Department of Pharmacognosy, Faculty of Pharmacy, Medical University, 1000 Sofia, Bulgaria
[2] Physiology and Biochemistry Research Laboratory, Department of Biology, Science Faculty, Selcuk University, Konya 42130, Turkey
* Correspondence: rgevrenova@pharmfac.mu-sofia.bg

Supplemental material

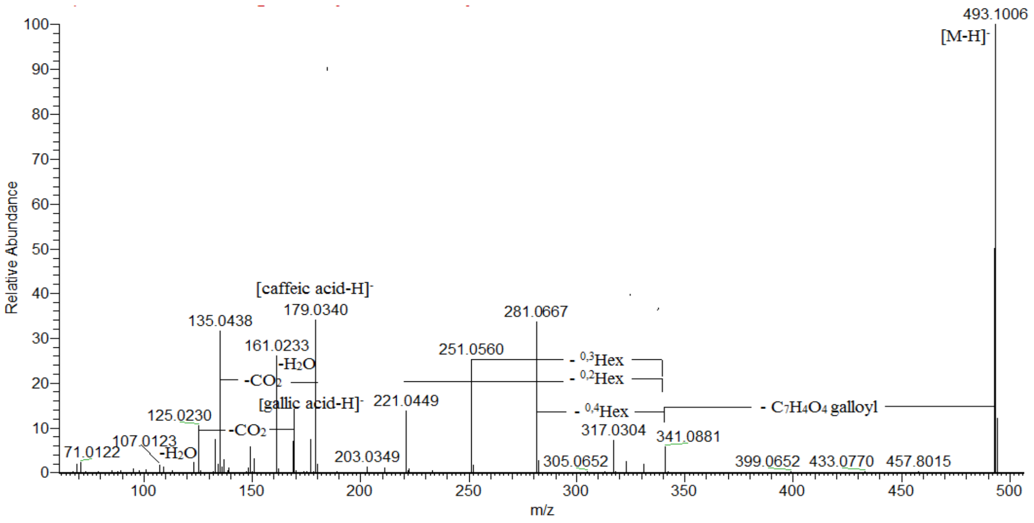

Figure S1. (-) ESI-MS/MS spectrum of galloyl-(caffeoyl)-hexose (25).

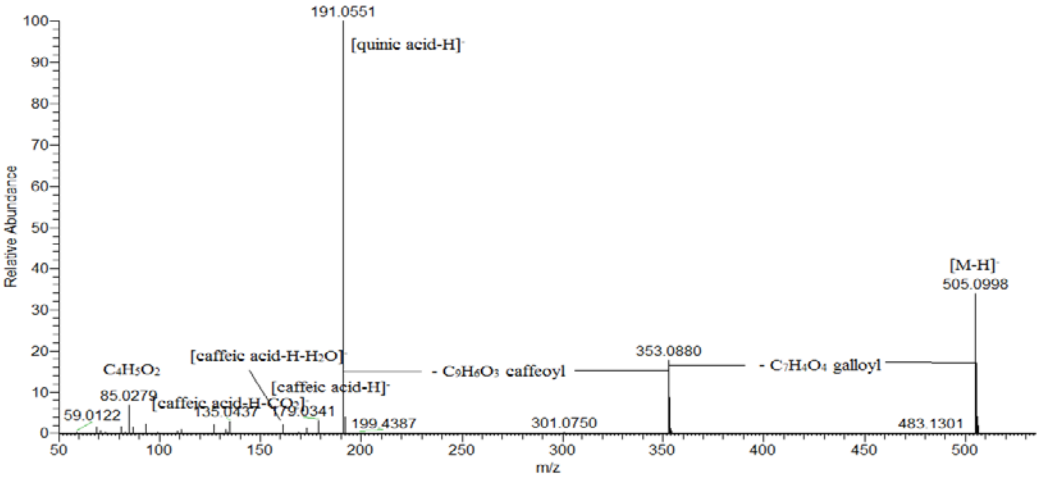

Figure S2. (-) ESI-MS/MS spectrum of 1-galloyl-5-caffeoylquinic acid (**38**).

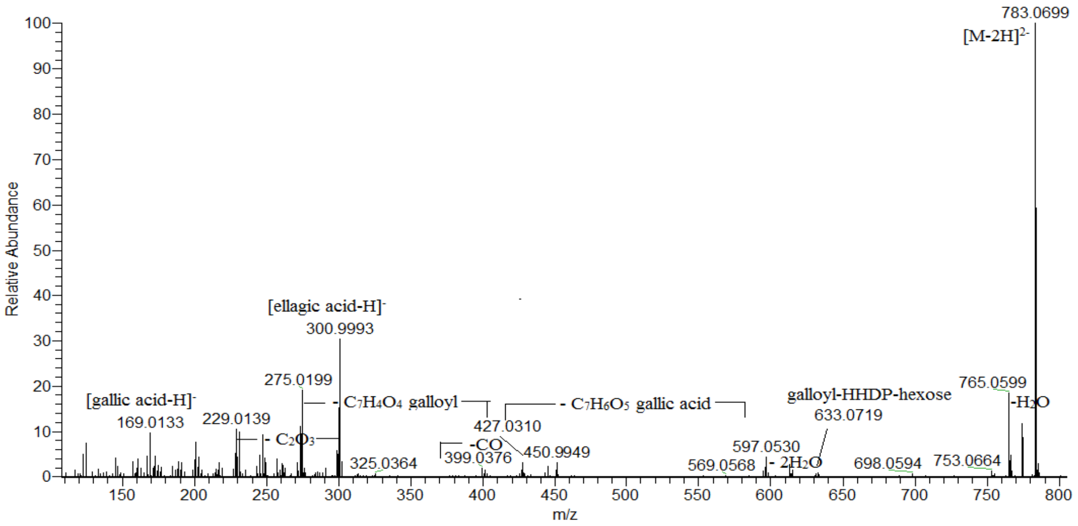

Figure S3. (-) ESI-MS/MS spectrum of oenothein B (50).

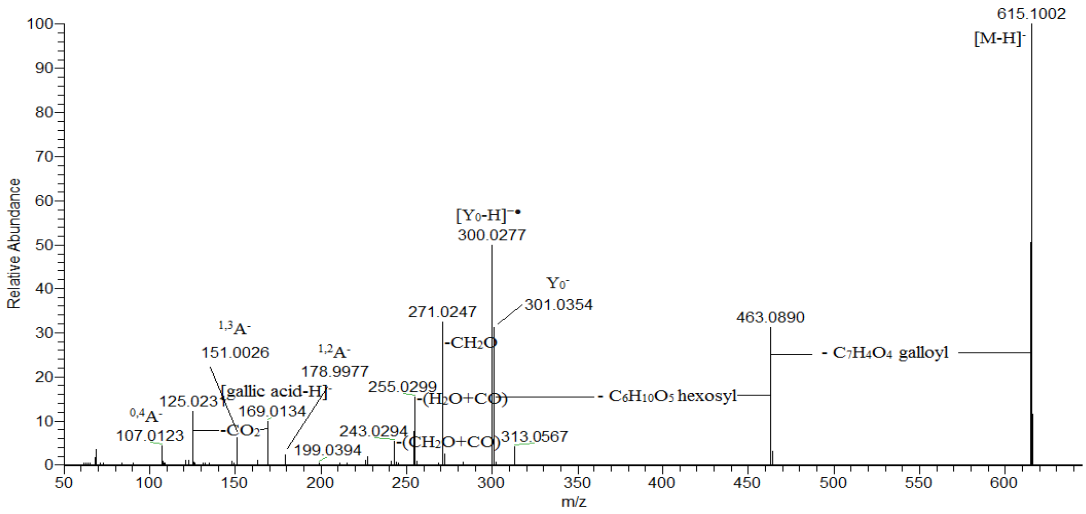

Figure S4. (-) ESI-MS/MS spectrum of quercetin 3-O-galloylhexoside (**79**).

Determination of Antioxidant and Enzyme Inhibitory Effects

Antioxidant (DPPH and ABTS radical scavenging, reducing power (CUPRAC and FRAP), phosphomolybdenum and metal chelating (ferrozine method)) and enzyme inhibitory activities (cholinesterase (Elmann's method), tyrosinase (dopachrome method), α-amylase (iodine/potassium iodide method), α -glucosidase (chromogenic PNPG method) and pancreatic lipase (p-nitrophenyl butyrate (p-NPB) method) were determined.

For the DPPH (1,1-diphenyl-2-picrylhydrazyl) radical scavenging assay: Sample solution was added to 4 mL of a 0.004% methanol solution of DPPH. The sample absorbance was read at 517 nm after a 30 min incubation at room temperature in the dark. DPPH radical scavenging activity was expressed as trolox equivalent (mg TE/g) (Kirby & Schmidt, 1997).

For ABTS (2,2′-azino-bis(3-ethylbenzothiazoline) 6-sulfonic acid) radical scavenging assay: Briefly, ABTS+ was produced directly by reacting 7 mM ABTS solution with 2.45 mM potassium persulfate and allowing the mixture to stand for 12–16 in the dark at room temperature. Prior to beginning the assay, ABTS solution was diluted with methanol to an absorbance of 0.700 ± 0.02 at 734 nm. Sample solution was added to ABTS solution (2 mL) and mixed. The sample absorbance was read at 734 nm after a 30 min incubation at room

temperature. The ABTS radical scavenging activity was expressed as trolox equivalent (mg TE/g) (Re et al., 1999).

For CUPRAC (cupric ion reducing activity) activity assay: Sample solution was added to premixed reaction mixture containing CuCl2 (1 mL, 10 mM), neocuproine (1 mL, 7.5 mM) and NH4Ac buffer (1 mL, 1 M, pH 7.0). Similarly, a blank was prepared by adding sample solution (0.5 mL) to premixed reaction mixture (3 mL) without CuCl2 . Then, the sample and blank absorbances were read at 450 nm after a 30 min incubation at room temperature. The absorbance of the blank was subtracted from that of the sample. CUPRAC activity was expressed as trolox equivalent (mg TE/g) (Apak, Güçlü, Özyürek, & Karademir, 2004).

For FRAP (ferric reducing antioxidant power) activity assay: Sample solution was added to premixed FRAP reagent (2 mL) containing acetate buffer (0.3 M, pH 3.6), 2,4,6-tris(2-pyridyl)-S-triazine (TPTZ) (10 mM) in 40 mM HCl and ferric chloride (20 mM) in a ratio of 10:1:1 (v/v/v). Then, the sample absorbance was read at 593 nm after a 30 min incubation at room temperature. FRAP activity was expressed as trolox equivalent (mg TE/g) (mg/ml) (Benzie & Strain, 1996).

For phosphomolybdenum method: Sample solution was combined with 3 mL of reagent solution (0.6 M sulfuric acid, 28 mM sodium phosphate and 4 mM ammonium molybdate). The sample absorbance was read at 695 nm after a 90 min incubation at 95 °C. The total antioxidant capacity was expressed as trolox equivalent (mmol TE/g) (Prieto, Pineda, & Aguilar, 1999).

For metal chelating activity assay: Briefly, sample solution was added to FeCl2 solution (0.05 mL, 2 mM). The reaction was initiated by the addition of 5 mM ferrozine (0.2 mL). Similarly, a blank was prepared by adding sample solution (2 mL) to FeCl2 solution (0.05 mL, 2 mM) and water (0.2 mL) without ferrozine. Then, the sample and blank absorbances were read at 562 nm after 10 min incubation at room temperature. The absorbance of the blank was subtracted from that of the sample. The metal chelating activity was expressed as EDTA equivalent (mg EDTAE/g) (mg/ml) (Dinis, Madeira, & Almeida, 1994).

For Cholinesterase (ChE) inhibitory activity assay: Sample solution (was mixed with DTNB (5,5-dithio-bis(2-nitrobenzoic) acid, Sigma, St. Louis, MO, USA) (125 μL) and AChE (acetylcholinesterase (Electric ell acetylcholinesterase, Type-VI-S, EC 3.1.1.7,Sigma)), or BChE (butyrylcholinesterase (horse serum butyrylcholinesterase, EC 3.1.1.8, Sigma)) solution (25 μL) in Tris–HCl buffer (pH 8.0) in a 96-well microplate and incubated for 15 min at 25 °C. The reaction was then initiated with the addition of acetylthiocholine iodide (ATCI, Sigma) or butyrylthiocholine chloride (BTCl, Sigma) (25 μL). Similarly, a blank was prepared by adding sample solution to all reaction reagents without enzyme (AChE or BChE) solution. The sample and blank absorbances were read at 405 nm after 10 min incubation at 25 °C. The absorbance of the blank was subtracted from that of the sample and the cholinesterase inhibitory activity was expressed as galantamine equivalent (mg GALAE/g) (Ellman, Courtney, Andres Jr, & Featherstone, 1961).

For Tyrosinase inhibitory activity assay: Sample solution was mixed with tyrosinase solution (40 μL, Sigma) and phosphate buffer (100 μL, pH 6.8) in a 96-well microplate and incubated for 15 min at 25 °C. The reaction was then initiated with the addition of L-DOPA (40 μL, Sigma). Similarly, a blank was prepared by adding sample solution to all reaction reagents without enzyme (tyrosinase) solution. The sample and blank absorbances were read at 492 nm after a 10 min incubation at 25 °C. The absorbance of the blank was subtracted from that of the sample and the tyrosinase inhibitory activity was expressed as kojic acid equivalent (mg KAE/g) (Masuda, Yamashita, Takeda, & Yonemori, 2005).

For α-amylase inhibitory activity assay: Sample solution was mixed with α-amylase solution (ex-porcine pancreas, EC 3.2.1.1, Sigma) (50 μL) in phosphate buffer (pH 6.9 with 6 mM sodium chloride) in a 96-well microplate and incubated for 10 min at 37 °C. After pre-incubation, the reaction was initiated with the addition of starch solution (50 μL, 0.05%). Similarly, a blank was prepared by adding sample solution to all reaction reagents without enzyme (α-amylase) solution. The reaction mixture was incubated 10 min at 37 °C.

The reaction was then stopped with the addition of HCl (25 µL, 1 M). This was followed by addition of the iodine-potassium iodide solution (100 µL). The sample and blank absorbances were read at 630 nm. The absorbance of the blank was subtracted from that of the sample and the α-amylase inhibitory activity was expressed as acarbose equivalent (mmol ACAE/g) (Šafašík, 1990).

For α-glucosidase inhibitory activity assay: Sample solution was mixed with glutathione (50 µL), α-glucosidase solution (from Saccharomyces cerevisiae, EC 3.2.1.20, Sigma) (50 µL) in phosphate buffer (pH 6.8) and PNPG (4-N-trophenyl-α-D-glucopyranoside, Sigma) (50 µL) in a 96-well microplate and incubated for 15 min at 37 °C. Similarly, a blank was prepared by adding sample solution to all reaction reagents without enzyme (α-glucosidase) solution. The reaction was then stopped with the addition of sodium carbonate (50 µL, 0.2 M). The sample and blank absorbances were read at 400 nm. The absorbance of the blank was subtracted from that of the sample and the α-glucosidase inhibitory activity was expressed as acarbose equivalent (mmol ACAE/g) (Ting, Zhang, Song, & Liu, 2005).

Porcine pancreatic lipase (type-II) activity was performed using *p*-nitrophenyl butyrate (p-NPB) as substrate (Roh and Jung, 2012). Enzyme solution (1 mg/mL) was prepared in 50 mM Tris-HCl (pH 8.0). Fraction solution (25 µL) was mixed with a lipase solution (50 µL) in a 96-well microplate and incubated for 20 minutes at 25°C. The reaction was initiated with the addition of p-NPB (5 mM, 50 µL). Similarly, a blank sample (prepared in the same manner but without the extract) was prepared for each of the samples and analysed according to this procedure. Milligrams of orlistat equivalents per gram of dry extract (OEs/g extract) were the measure unit.

References

Apak, R., Güçlü, K., Özyürek, M., & Karademir, S. E. (2004). Novel total antioxidant capacity index for dietary polyphenols and vitamins C and E, using their cupric ion reducing capability in the presence of neocuproine: CUPRAC method. Journal of agricultural and food chemistry, 52(26), 7970-7981.

Arvouet-Grand, A., Vennat, B., Pourrat, A., & Legret, P. (1994). [Standardization of propolis extract and identification of principal constituents]. J Pharm Belg, 49(6), 462-468.

Benzie, I. F., & Strain, J. J. (1996). The ferric reducing ability of plasma (FRAP) as a measure of "antioxidant power": the FRAP assay. Analytical biochemistry, 239(1), 70-76.

Dinis, T. C., Madeira, V. M., & Almeida, L. M. (1994). Action of phenolic derivatives (acetaminophen, salicylate, and 5-aminosalicylate) as inhibitors of membrane lipid peroxidation and as peroxyl radical scavengers. Archives of biochemistry and biophysics, 315(1), 161-169.

Ellman, G. L., Courtney, K. D., Andres Jr, V., & Featherstone, R. M. (1961). A new and rapid colorimetric determination of acetylcholinesterase activity. Biochemical pharmacology, 7(2), 88-95.

Kirby, A. J., & Schmidt, R. J. (1997). The antioxidant activity of Chinese herbs for eczema and of placebo herbs—I. Journal of ethnopharmacology, 56(2), 103-108.

Masuda, T., Yamashita, D., Takeda, Y., & Yonemori, S. (2005). Screening for tyrosinase inhibitors among extracts of seashore plants and identification of potent inhibitors from Garcinia subelliptica. Bioscience, biotechnology, and biochemistry, 69(1), 197-201.

Prieto, P., Pineda, M., & Aguilar, M. (1999). Spectrophotometric quantitation of antioxidant capacity through the formation of a phosphomolybdenum complex: specific application to the determination of vitamin E. Analytical biochemistry, 269(2), 337-341.

Re, R., Pellegrini, N., Proteggente, A., Pannala, A., Yang, M., & Rice-Evans, C. (1999). Antioxidant activity applying an improved ABTS radical cation decolorization assay. Free radical biology and medicine, 26(9-10),

1231-1237. Roh, C., Jung, U., 2012. Screening of crude plant extracts with anti-obesity activity. Int. J. Mol. Sci. 132, 1710-1719.

Šafašík, I. (1990). Rapid Detection of Alpha-Amylase Inhibitors. Journal of Enzyme Inhibition, 3(3), 245-247.

Slinkard, K., & Singleton, V. L. (1977). Total phenol analysis: automation and comparison with manual methods. American journal of enology and viticulture, 28(1), 49-55.

Ting, L., Zhang, X.-d., Song, Y.-w., & Liu, J.-w. (2005). A microplate-based screening method for alpha-glucosidase inhibitors. Chinese Journal of Clinical Pharmacology and Therapeutics, 10(10), 1128.
